# I understand your pain but I do not feel it: lower affective empathy in response to others’ social pain in narcissism

**DOI:** 10.3389/fpsyg.2024.1350133

**Published:** 2024-03-15

**Authors:** Fatemeh Shahri, Abbas Zabihzadeh, Alireza Taqipanahi, Morteza Erfani Haromi, Mobina Rasouli, Asal Saeidi Nik, Clare M. Eddy

**Affiliations:** ^1^Department of Psychology, Shahid Behashti University, Tehran, Iran; ^2^Institute for Cognitive and Brain Sciences, Shahid Beheshti University, Tehran, Iran; ^3^College of Medical and Dental Sciences, University of Birmingham and Birmingham and Solihull Mental Health NHS Foundation Trust, Birmingham, United Kingdom

**Keywords:** affective empathy, cognitive empathy, empathic accuracy, narcissism, physical pain, self-other distinction, social pain

## Abstract

**Introduction:**

While the relationship between narcissism and empathy has been well-researched, studies have paid less attention to empathic accuracy, i.e., appreciating the precise strength of another person’s emotions, and self-other distinction, in terms of the disparity between affective ratings for self and other in response to emotive stimuli. Furthermore, empathic responses may vary depending on whether the pain is physical or social.

**Methods:**

We investigated empathic accuracy, affective empathy, and the distinction between pain, emotion and intensity ratings for self and other, in high (*n* = 44) and low (*n* = 43) narcissism groups (HNG and LNG, respectively) selected from 611 students, in response to both types of pain. Participants watched six videos where targets expressed genuine experiences of physical and social pain, and rated the perceived affect and pain experienced by the person in the video and their own empathic emotional responses.

**Results and discussion:**

The HNG displayed lower affective empathy and empathic accuracy than the LNG for both pain types. Within the HNG there was higher empathic accuracy for social vs. physical pain, despite reduced affective empathy for social pain, in contrast to the LNG. In addition to this paradox, the HNG demonstrated greater differences between ratings for the self and for target others than the LNG, suggesting that narcissism is associated with higher self-other distinction in response to viewing other people describing social pain.

## Introduction

Narcissism is a dark personality trait (Smith et al., 2018), and its clinical manifestation, narcissistic personality disorder, is characterized by behavioral and thinking patterns including entitlement, a sense of superiority and uniqueness, excessive need for admiration, self-centeredness, and altered empathy (Bukowski and Samson, 2021; Eddy, 2021; Gruda et al., 2021). People with narcissistic traits have a hard time maintaining relationships (Back et al., 2013; Hart et al., 2018). Even though they may seem attractive to potential partners (Back et al., 2010), incompatible characteristics such as the strong desire for power and revenge (Wilson et al., 2017), betrayal and lack of commitment (Wurst et al., 2017), unwillingness to apologize (Leunissen et al., 2017), aggression (Hyatt et al., 2019), and a tendency to objectify others (Lachowicz-Tabaczek et al., 2021) can become apparent over time, leading to interpersonal difficulties (Campbell and Campbell, 2009). Despite ongoing debates regarding the definition and concept of trait narcissism (Miller et al., 2017), there is a consensus among researchers that it encompasses multiple dimensions, each with distinct forms or aspects (Krizan and Herlache, 2018) and varying implications for human behavior and well-being (Duffner et al., 2019). A critical and increasingly recognized distinction within the field is between grandiose and vulnerable narcissism (Wink, 1991). Grandiose narcissism is generally characterized by self-centered self-exaltation (Morf and Rhodewalt, 2001), aligning closely with everyday perceptions of narcissism, whereas vulnerable narcissism is associated with feelings of insufficiency and incompetence (Miller et al., 2011). The primary focus of this study was on the grandiose subtype of narcissism.

One critical feature of the narcissistic personality structure that may contribute to interpersonal difficulties is a reduced tendency to empathize with others (Hart et al., 2018). Empathy refers to the ability to understand and share the feelings and intentions of others (Decety and Jackson, 2004) and has two components, affective and cognitive (Decety and Jackson, 2004; Shamay-Tsoory et al., 2009). Whereas affective empathy (AE) is the emotional response within an individual that mirrors the emotional state of another person (Eisenberg and Miller, 1987), cognitive empathy (CE), is the capacity to recognize and understand the emotions, thoughts, and intentions of others (Davis, 1980; Bos and Stokes, 2019). CE can be measured in terms of empathic accuracy: the ability to accurately understand another person’s thoughts and emotions (Zaki and Ochsner, 2011). This concept is often measured by comparing the emotions and thoughts an individual reports with the inference made by another person observing them (Zaki and Ochsner, 2011).

### Empathy and narcissism

Whereas research focusing on narcissism invariably reports a decline in AE, the results are less clear when it comes to CE (e.g., Wai and Tiliopoulos, 2012; Vonk et al., 2013; Pajevic et al., 2018; Turner et al., 2019; di Giacomo et al., 2023). Jonason and Krause (2013) observed that narcissism correlates with decreased affective empathy and difficulties in emotion recognition. However, more recent studies suggest that whereas narcissism negatively impacts affective empathy, it might be positively linked with cognitive empathy (Turner et al., 2019; Doyle, 2020; Wertag et al., 2021; Duradoni et al., 2023). One contributing factor to these inconsistencies is measurement methods, which range from self-report assessments to behavioral tasks. Data from different tasks show that empathy in narcissism is not solely characterized by deficiency but can also vary due to motivational factors (Baskin-Sommers et al., 2014; Hepper et al., 2014a; Jacobs, 2022). Pajevic et al. (2018) found that despite higher self-report cognitive empathy among narcissistic individuals, this did not translate to better performance in emotion recognition tasks, which showed a nonsignificant correlation. Additionally, a meta-analysis by Urbonaviciute and Hepper (2020) indicated that grandiose aspect of narcissism correlates negatively with AE and CE based on self-report measures. Similarly, objective, performance-based measures echoed this negative trend between grandiosity and AE. However, when CE was evaluated using performance-based measures, no clear relationship was found with this subtype of narcissism.

### Physical and social pain

Empathic responses have been widely studied through observing another individual’s suffering, which can be caused by either physical (Benuzzi et al., 2008; Avenanti et al., 2010) or social (Zaki et al., 2009) pain stimuli. Whereas physical pain is associated with bodily injury, social pain refers to discomfort caused by the possible or actual loss of social bonds (Eisenberger, 2011), e.g., ostracism, betrayal, or rejection (Riva et al., 2016). Social and physical pain share commonalities in terms of evolutionary function (MacDonald and Leary, 2005) and involve partial overlapping brain circuitry (DeWall et al., 2010). Furthermore, both forms of pain can elicit fear and anxiety (Riva et al., 2014a,b), and lead to comparable psychological outcomes (Riva et al., 2011, 2014a,b). Whereas several studies have explored the disparities in the perception of these two types of pain in personal experiences (Eisenberger, 2011; Brunell et al., 2021), recent research has expanded to include how observers perceive and evaluate physical vs. social pain experienced by others (Riva and Andrighetto, 2012; Riva et al., 2014a,b, 2016; Atkins et al., 2016) as well as their empathetic responses to these types of pain (Flasbeck et al., 2017). In relation to the observation of pain experienced by others, understanding can be influenced by multiple factors, including racial differences (Avenanti et al., 2010; Riva and Andrighetto, 2012), gender (Riva et al., 2011), moral judgments (Riva et al., 2016), cultural background (Atkins et al., 2016), and the personality of both the observer and the person experiencing pain (Flasbeck et al., 2017). Given the inevitable nature of social pain within interpersonal relationships and its more lasting impact compared to physical pain (Chen et al., 2008), it is crucial to examine the differences in how individuals with grandiose narcissism respond to both physical and social pain stimuli. These people might suppress negative emotions related to social pain as a strategy to avoid feelings of failure, which are more prevalent in social contexts, thus experiencing this type of pain less intensely (Brunell et al., 2021). The current study explored empathic reactions toward the social and physical pain of others among individuals with grandiose narcissistic traits, given that grandiose narcissism might differentially influence the ability to recognize, accurately evaluate, and empathize with the physical and social pain experienced by others, suggesting that their diminished empathetic responses are notably more evident when confronting the social pain of others rather than physical pain.

### Self-other distinction

The experience of empathy toward another’s pain appears to provoke neural responses similar to those we undergo when feeling pain personally (Singer et al., 2004; Riečanský et al., 2020), suggesting that we employ our own emotional systems to interpret and empathize with what another individual is experiencing and feeling. This is closely tied to the concept known as self-other distinction, i.e., our ability to differentiate between our own mental and physical states and those of others. Self-other distinction describes an intraindividual concept or experience, but it is likely to be closely linked to empathy and social behavior. Therefore while self-other distinction may be applied to different domains (e.g., motor, cognitive, and emotional: see Eddy, 2022), measuring self-other distinction may appear to overlap with affective empathy, when applied to emotional experience. For example, when self-other distinction within an individual is less pronounced, it may facilitate greater affective empathy or manifest as greater resonance with the emotion of another, such as experiencing personal distress in response to another person’s distress (e.g., Eddy, 2022). Intact CE in the context of lower AE, and many characteristics associated with narcissism, such as socially competitive emotions, seem to imply higher self-other distinction in highly narcissistic individuals (Eddy, 2022). However, self-other distinction may vary according to the subtype of narcissism, as one study (De Panfilis et al., 2019) found high personal distress (which may indicate low self-other distinction) in individuals with narcissistic traits who experience rejection sensitivity (suggestive of vulnerable narcissism) Consequently, the current study explored self-other distinction in individuals with more grandiose narcissistic traits, aiming to deepen our understanding of how this construct varies across narcissism subtypes.

### Hypotheses and aims

Previous research exploring pain and empathy in those with narcissistic traits has not distinguished between physical and social pain. However, Brunell et al. (2021) explored the self-experience of these two types of pain in individuals with narcissism. Their research revealed that although grandiose narcissism is not directly associated with the sensation of physical pain, it is linked to a negative emotional response when experiencing it. Conversely, grandiose narcissistic individuals reported minimal impact in response to social pain and remained focused on cognitive tasks. This pattern of behavior arises from their tendency to place the blame for social discomfort on others, justifying their own innocence by adopting an “it’s them, not me” attitude (Brunell et al., 2021). Whereas this attitude cannot alleviate the sensation of physical pain, it plays a crucial role in diminishing the experience of social pain. Therefore, this difference in processing their own social pain relative to physical pain may reflect differential empathic responses to the social and physical pain of others. Based on these observations, the current study aimed to explore both empathy for physical and social pain in narcissism, and we expected that individuals with high levels of grandiose narcissism would exhibit lower affective empathy in response to social pain when compared to physical pain.

Given that self-report methods largely gauge motivation to empathize rather than actual capacity (Urbonaviciute and Hepper, 2020), we chose a video-task to assess empathy behaviorally. This approach provides a more ecologically valid measure of narcissists’ empathic behavior, which may be a better predictor of actual interpersonal skills. Due to the connection between affective empathy and self-other distinction (i.e., greater affective empathy may imply lower self-other distinction), and the possible tendency of individuals with grandiose narcissism to distance themselves to avoid engagement with others’ emotions (thereby lacking affective empathy), we were motivated to draw upon these experimental measures in order to explore self-other distinction. We expected that individuals with higher levels of grandiose narcissism would exhibit lower affective empathy, and greater self-other distinction (Eddy, 2022) than less narcissistic individuals. Taking our two hypotheses together, we also expected that the difference in self-other distinction between individuals with high versus low narcissism would be greater for social than physical pain stimuli.

## Methods

### Participants and procedure

The ethics committee of the Faculty of Psychology and Education of Shahid Beheshti University approved the procedure (IR.SBU.REC.1402.044). All participants gave written informed consent. The study involved two parts: the creation of physical and social pain stimuli, and then the testing of two subgroups of participants who had been screened and sub-grouped based on level of narcissism. Firstly, volunteers (i.e., targets) from the Faculty of Psychology at Shahid Behehsti University were recruited to form the stimulus creation sample, and provided ratings of pain, affect, and intensity for comparison to participants. To ensure targets were not familiar to participants who rated the videos, those participants were recruited from a separate student sample, divided into high and low narcissism groups, from other faculties at the University. Participants were paid to take part in the 40 min procedure, which included rating the degree of pain and affect experienced by the target and themselves in response to the video. They also completed measures to assess baseline emotional state, mental and physical health, and self-report empathy, described below.

The pool used to create high and low narcissism groups consisted of 611 students (337 females; M_age_ = 22.4, SD = 4.6). We used the Persian version (Mohammadzadeh, 2009) of the Narcissistic Personality Inventory-16 (NPI-16; Ames et al., 2006) to evaluate subclinical narcissism. The total score ranges from 0 to 16 and is positively associated with narcissism, focusing on more grandiose traits. There are no categories or cut-off points on the scale (Raskin and Hall, 1981; Raskin and Terry, 1988). The average NPI-16 score in the testing pool was 6.08 (SD = 3.08). The cut-off was mean + 1SD for the high narcissism group (“HNG”; *N* = 79, 32 females; M_age_ = 21.76, SD = 3.54; M_NPI-16_ = 11.23, SD = 1.39, Range = 10–16), and mean-1SD for the low narcissism group (“LNG”; *N* = 71, 48 females; M_age_ = 23.25, SD = 5.87; M_NPI-16_ = 1.51, SD = 0.65, Range = 0–2). Prior to the study, we determined to gather data from at least 100 participants—50 participants per group, using an established rule of thumb (see Nelson et al., 2018). From these two groups, 49 participants from the LNG and 50 from the HNG agreed to participate in the study. Participants who met any of the following criteria were excluded: current diagnosis of a psychiatric or neurological disorder, experiencing any physical or social pain at the time of assessment, taking medications that affect autonomic arousal or pain experience (e.g., mood stabilizers, analgesics), and exhibiting severe clinical levels of depression, anxiety, and stress, as assessed by the Depression and anxiety stress scale 21 (DASS-21) questionnaire (Lovibond and Lovibond, 1995). In total, 12 participants were excluded based on these criteria (two due to recent relationship breakdown, two due to medication usage, one diagnosed with bipolar disorder, four for depression, two for anxiety, and one for stress), resulting in a final sample of 87 participants, with 44 (15 females; M_age_ = 21.6, SD = 3.6; M_NPI-16_ = 11.27, SD = 1.59, Range = 10–16) classified as highly narcissistic and 43 (30 females, M_age_ = 21.7, SD = 4.1; Mean_NPI-16_ = 1.55, SD = 0.7, Range = 0–2) as low narcissistic. Following these exclusions, we confirmed that no extreme outliers were present in our data. Our sample scores were found to be consistent with previous studies (e.g., Casale et al., 2016; Aytaç and Akın, 2021) and all participants in the HNG scored above the previously suggested cut-off score (8) likely to indicate Narcissistic Personality Disorder (Vaziri-Harami et al., 2021). Sensitivity analyses using G*Power Version 3.1.9 (Faul et al., 2009) indicated our sample size to be sufficiently powered at 80% for detecting medium-sized within-between subject effects in analysis of variance (ANOVA) models (Cohen’s *f* = 0.15).

#### Phase 1: pain stimuli creation and target ratings

To create physical and social pain stimuli, we followed the protocol used by other researchers (Zaki et al., 2009; Atkins et al., 2016; Jospe et al., 2020). We invited 16 volunteers (nine females; M_age_ = 22.62, SD = 1.78) from the stimulus creation sample to the lab. We filmed them while they explained a physically or socially painful experience. Some who had experienced both types of pain were filmed twice (for physical pain, and social pain). These targets first completed the 16-item Berkeley Expressivity Questionnaire (BEQ; see Gross et al., 2000), which measures how much individuals believe their emotions are visible to others (e.g., “Whenever I feel negative emotions, people can easily see exactly what I am feeling”). This ensured uniformed expressivity across individuals explaining both social and physical pain stimuli. Following that, they were settled in a quiet room where they spent around 5 min reflecting on the pain they had experienced. To facilitate better memory retrieval, we asked them to give each story a title and write a short description of the incident. After that, we positioned the camera in front of them to capture their upper body. Once ready, we left the room, and they began describing their personal experience of pain in front of the camera, sharing their feelings and reflections for approximately 2–3 min. Immediately after the recording, targets watched their videos and rated the intensity (1 = not intense at all to 9 = extremely intense) and affective valence (1 = extremely negative to 9 = extremely positive) of the actual recall experience in the video, which were later used for video selection. They also completed the short form of Rejected Emotion Scale (Buckley et al., 2004), using a nine-point Likert scale ranging from (1 = not at all to 9 = extremely). They indicated the extent of pain and sadness, hurt feelings, anxiety, happiness, and anger experienced while recalling the physically or socially painful event. Targets were explicitly instructed to evaluate the affect they experienced while describing the pain rather than during the actual event or when watching the video replay (Jospe et al., 2020). They provided informed consent before the recording and again after recording to permit the use of their video in research.

A total of 26 videos were recorded involving 16 targets. Half captured physical pain experiences, and the other half social pain. The selection of videos for the current study was based on the following criteria; one video was selected from each target, and four videos were excluded for being shorter than 2–3 min. The resulting 12 videos included six for each type of pain. Next, ratings given by the targets were used to balance intensity and valence for each category, leaving 10 videos. The final selection of six videos was made to further ensure consistent video topic and ease of comprehension (Atkins et al., 2016). The social pain videos described losing a loving father, being ignored by a best friend, and being lonely. The physical pain stimulus videos described sustaining a cruciate ligament injury, a toe injury, and accidentally cutting fingers with a grate. Each video underwent editing in Adobe Premiere 6 software to remove background noise, standardize frame sizes, and incorporate the same opening sequence between them (see Table 1).

**Table 1:** Demographic variables and self-report measure scores for selected video stimulus.

	Social pain videos (*n* = 3)	Physical pain videos (*n* = 3)	*p*
Gender (Male/Female)	2/1	2/1	1
Age (Years)	24 ± 2	23.3 ± 2.3	0.72
Education (Years)	17.3 ± 1.15	16.6 ± 1.15	0.51
Duration of the videos (Sec)	128.6 ± 10.7	138.3 ± 31.7	0.64
Affective valence (rated by targets)^a^	2 ± 1	2.66 ± 0.57	0.37
Intensity (rated by targets)^b^	6.33 ± 0.57	7.33 ± 0.57	0.10
Berkeley expressivity questionnaire score	5.23 ± 0.72	4.98 ± 0.77	0.70

*p* values reflect the level of significance from independent samples *t*-test and chi-square as appropriate. Values are given in mean ± SD. ^a^1 = extremely negative to 9 = extremely positive. ^b^1 = not intense at all to 9 = intensive the most.

#### Phase 2: observer ratings

**Figure 1:**
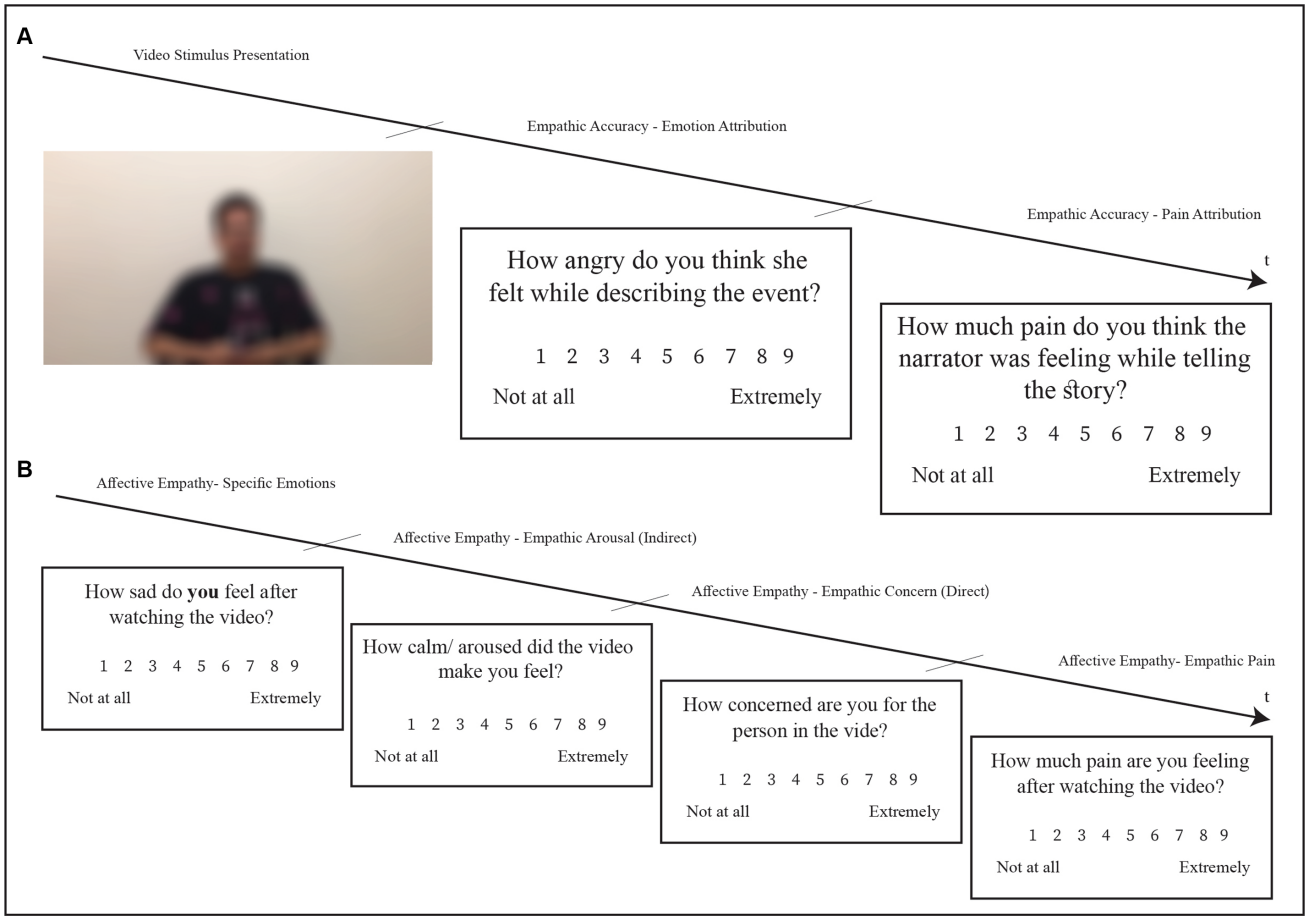
Rating process for each video completed by participants in high narcissism group (HNG) and low narcissism group (LNG). **(A)** Screen shots of the observers’ others-rating phase (Emotion Attribution and pain attribution). **(B)** Screen shots of the observers’ self-rating phase (Components of Affective Empathy).

All study participants from the LNG and HNG observed and rated the pain videos at the Psychology lab after completing the *Positive and Negative Affect Schedule* (PANAS; Watson et al., 1988) to establish parity of baseline emotional state across the groups. Next, they watched the six videos individually, in random order (using Psychopy 2020.2.4 software; Peirce et al., 2019), before rating the pain and intensity of each emotion the target in the video was feeling, using the same items and scales (i.e., for pain and specific emotions) as targets, in order to assess empathic accuracy (emotion and pain attribution). They provided self-ratings of their degree of pain (empathic pain) and concern (empathic concern) they felt toward the person in the video, as well as their own feelings (specific emotions: sadness; hurt; anxiety; happiness; and anger) and level of arousal (empathic arousal), to measure explicit and implicit affective empathy, respectively (see Dziobek et al., 2008; Figure 1). Participants also completed the *Interpersonal Reactivity Index* (IRI; Davis, 1980) to provide a general assessment of self-reported trait empathy.

### Self-report measures completed by participants in the HNG and LNG

#### Positive and negative affect schedule

The Positive and Negative Affect Schedule (PANAS) is a self-report measure that assesses general affect (Watson et al., 1988). Comprising 20 items, it has two subscales, each with 10 words describing positive (*α* = 0.88) and negative (*α* = 0.87) emotions. Respondents evaluate each item using a scale from 1 (“Very slightly or not at all”) to 5 (“Extremely”). The ratings from these items were averaged to generate separate positive and negative affect measures.

#### Interpersonal reactivity index

This self-report questionnaire assesses empathy’s cognitive and affective components as separate constructs (Davis, 1980). In this study, we utilized the Persian Version of the short form of the IRI, validated by Golbabaei et al. (2023). The scale consists of four subscales, each containing four items: Empathic Concern (*α* = 0.67) and Personal Distress (*α* = 0.71) measure affective empathy, whereas Perspective Taking (*α* = 0.67) and Fantasy (*α* = 0.69) assess cognitive empathy.

#### Depression and anxiety stress scale 21

The DASS-21 is self-report and contains three subscales, each with seven items: depression (*α* = 0.81), anxiety (*α* = 0.73), and stress (*α* = 0.81) (Lovibond and Lovibond, 1995). Participants were asked to rate these items on a four-point scale, ranging from 0 (“did not apply to me at all”) to 3 (“applied to me very much, or most of the time”). Scores for each component were calculated by summing the responses for individual items within each subscale. In this study, participants in both groups were excluded if their scores exceeded the normal range, defined as >14 for depression, >12 for anxiety, and > 17 for stress. The normal range values for the subscales were determined based on the Iranian version of the DASS-21, validated by Sahebi et al. (2005).

### Statistical analysis

#### Scoring procedure for empathic accuracy-emotion attribution

We used a similar procedure to Jospe et al. (2020) to compute empathic accuracy scores. Firstly, distance was calculated between the participant’s ratings for the target and the target’s self-ratings for each of the five emotions on a nine-point scale (1–9). Scores were subtracted then reverse scored, e.g., a difference of 8 was scored 0; a difference of 0 was scored 8. Therefore, the range of possible distance scores extended from 0 to 40. Secondly, recognition score was assigned if the presence or absence of a specific emotion was accurately identified. Thus, if the participant gave a rating of 1 for a particular emotion (they believed the emotion was not present), and the target rated it 2 or higher (it was present), the participant would gain a 0, indicating they did not correctly identify the presence of the emotion. If they provided a rating of 2 or more, they would receive a score of 1, demonstrating successful emotion recognition. The same rule was applied when the target emotion was absent. The score of this measure was the proportion of correct recognition out of all 5 emotions, so the highest score would be 5/5 (1), representing perfect recognition, whereas the lowest score is 0/5 (0), indicating no correct recognition. The overall score for empathic accuracy was obtained by multiplying the distance score by the recognition score.

Demographic variables (gender, age, and year of study), trait empathy (IRI-16), and general affect (PANAS) were compared for the high and low narcissism groups using independent samples *t*-tests and chi-square. To examine whether the HNG and LNG differed in terms of empathic accuracy (measured by emotion attribution and pain attribution), affective empathy (measured by specific emotions, empathic concern, empathic arousal, and empathic pain), and self-other distinction in response to watching social pain and physical pain videos, we conducted a series of 2 × 2 repeated measures ANOVA with each outcome measure as dependent variables, trait narcissism group (high vs. low) as the between-subjects variable, and pain type (physical vs. social) as the within-subjects variable (see Table 2 for descriptive statistics).

**Table 2:** Demographic variables and self-report measure scores for HNG (*n* = 43) and LNG (*n* = 43).

	High narcissistic group (*n* = 44)	Low narcissistic group (*n* = 43)	*p*
Gender (Male/Female)	29/15	13/30	0.001
Age (Years)	21.66 ± 3.64	21.77 ± 4.14	0.897
NPI scores	11.27 ± 1.59	1.55 ± 0.7	0.000
Education (Years)	15.02 ± 2.01	15.16 ± 2.13	0.754
Affective empathy (IRI)			
Personal distress	2.07 ± 0.79	2.57 ± 0.73	0.003
Empathic concern	2.51 ± 0.80	3.08 ± 0.49	0.000
Cognitive empathy (IRI)			
Perspective taking	2.64 ± 0.82	2.54 ± 0.70	0.563
Fantasy	2.31 ± 0.82	2.52 ± 0.74	0.214
General affect (PANAS)			
Positive affect	3.61 ± 0.65	2.93 ± 0.63	0.000
Negative affect	2.24 ± 0.65	2.27 ± 0.69	0.855

*p* values reflect the level of significance from independent samples *t*-test and chi-square as appropriate. Values are given in mean ± SD.

Comparisons specifically relevant to self-other distinction included within participant (1) differences between attributions of pain and emotions to the target vs. ratings of self-emotions and pain in response to the videos; and (2) the difference between the targets’ ratings of their pain/emotions/intensity during recording compared to the pain/emotion/arousal felt by observers (i.e., pain/emotional/intensity resonance), with more similar ratings for participant and target suggesting lower self-other distinction. Therefore, whereas our assessment of self-other distinction relied upon the same tasks used to assess affective empathy and empathic accuracy, this construct was explored in a distinct way by using specific within participant comparisons between numerous ratings for the self vs. the other. This first measure (1, above) was the primary measure of self-other distinction because it involved within-participant ratings. The resonance measures (2, above) are more similar to affective empathy and empathic accuracy as they compare ratings between targets and participants, but they are calculated differently to those measures. We would expect these latter measures to be closely tied to the within-participant rating of self-other distinction given that, e.g., feelings of greater separation from the other should be more likely when there is lower resonance with the other’s perceived state. However, they less directly reflect the current conceptualization of self-other distinction as an intra-individual construct.

Bonferroni correction was used to compare groups in terms of the mean estimated in empathic responses toward physical and social pain in *post hoc* tests with corrected *p* values. Instances of non-sphericity in Mauchly’s Test, we used Greenhouse–Geisser corrected *p* values. Statistical analyses were done in SPSS-26. For all tests, a significance level of *p* < 0.05 was chosen.

## Results

### Between group differences

#### Demographic characteristics

T-tests indicated no differences between groups in age, *t*(85) = 0.13, *p* = 0.897, 95% CI [−1.554, 1.770] and education *t*(85) = 0.314, *p* = 0.754, 95% CI [− 0.746, 1.026]. Nevertheless, according to the chi-square test, significant gender differences were found between the HNG and LGN. Descriptive statistics are shown in Table 2.

#### Trait empathy (IRI scores)

##### Affective empathy

The HNG scored significantly lower than the LNG on the Empathic Concern, *t*(85) = 4.017, *p* < 0.001, 95% CI [0.29085, 0.86084] and Personal Distress, *t*(85) = 3.077, *p* = 0.003, 95% CI [0.17754, 0.82589] subscales of the IRI.

##### Cognitive empathy

T-tests indicated no between-group differences for perspective-taking, *t*(85) = − 0.581, *p* = 0.563, 95% CI [− 0.42239, 0.23133] or Fantasy *t*(85) = 1.252, *p* = 0.214, 95% CI [− 0.12430, 0.54581] subscales of the IRI.

#### General affect (PANAS scores)

##### Positive and negative affect

There were no differences between the HGN and LGN in negative affect, *t*(85) = 0.184, *p* = 0.855, 95% CI [− 0.26132, 0.31449]. However, the two groups significantly differed in the positive affect with higher scores in the HNG, *t*(85) = −4.925, *p* < 0.001, 95% CI [− 0.95925, − 0.40745].

### Video task results

#### Empathic accuracy

##### Emotion attribution

Narcissism had a significant main effect, *F*(1, 85) = 4.2, *p* = 0.043, ηp^2^ = 0.047, such that individuals in the LNG were significantly more empathically accurate compared to those in the HNG in both pain types. Furthermore, a significant main effect of pain type was found, *F*(1, 85) = 6.12, *p* = 0.015, ηp^2^ = 0.067, indicating that participants in both groups had higher empathic accuracy for social pain videos than physical pain ones. The group × pain type interaction was insignificant, *F* (1, 85) = 0.165, *p* = 0.686 (see Table 3 for descriptive statistics).

**Table 3:** Mean (SD) scores for component of empathic accuracy, affective empathy, and self-other distinction in response to physical and social pain separately for HNG and LNG.

Measure	High narcissistic group		Low narcissistic group	
	Physical pain	Social pain	Physical pain	Social pain
	M ± SD	M ± SD	M ± SD	M ± SD
Empathic accuracy − Pain attribution	5.89 ± 0.89	5.69 ± 0.80	5.93 ± 0.85	5.96 ± 0.65
Empathic accuracy − Emotion attribution	22.88 ± 4.94	24.10 ± 3.8	23.99 ± 4.44	25.70 ± 3.06
Affective empathy − Specific emotions	4.35 ± 1.08	4.62 ± 1.17	4.83 ± 1.16	5.62 ± 1.13
Affective empathy − Empathic arousal	4.68 ± 1.64	4.62 ± 1.71	5.42 ± 1.71	6.09 ± 1.52
Affective empathy − Empathic concern	4.77 ± 1.70	4.68 ± 1.77	5.17 ± 1.50	6.07 ± 1.64
Affective empathy − Empathic pain	5.29 ± 1.70	4.67 ± 1.87	5.48 ± 1.70	6.14 ± 1.42
Self-other distinction-within participants − Emotions	−4.38 ± 5.28	–7.67 ± 4.85	−3.14 ± 3.73	–4.06 ± 3.57
Self-other distinction-within participants − Pain	−0.54 ± 1.49	–1.47 ± 1.45	−0.86 ± 2.1	–0.54 ± 1.13
Self-other distinction − Emotion resonance	−5.26 ± 5.64	–5.79 ± 5.87	−3.65 ± 5.62	–1.6 ± 5.61
Self-other distinction − Pain resonance	−0.69 ± 1.71	–2.00 ± 1.88	−0.47 ± 1.75	–0.53 ± 1.42
Self-other distinction − Intensity resonance	−1.65 ± 1.64	–2.71 ± 1.71	−0.90 ± 1.72	–1.24 ± 1.52

##### Pain attribution

There was no significant main effect of narcissism on pain ratings, *F* (1, 85) = 1.35, *p* = 0.248. Additionally, there was no significant effect of pain type, *F* (1, 85) = 0.615, *p* = 0.435. The interaction effect between narcissism level x pain type was also insignificant, *F* (1, 85) = 1.134, *p* = 0.29, suggesting that both groups perceived pain at comparable levels for each type of pain.

#### Affective empathy

##### Specific emotions

There was a significant main effect of narcissism, *F*(1, 85) = 11.58, *p* = 0.001, ηp^2^ = 0.12, demonstrating that participants in the LNG had higher levels of affective empathy. There was also a significant main effect of pain type, *F*(1, 85) = 23.85, *p* < 0.001, ηp^2^ = 0.219, i.e., both groups showed greater affective empathy toward social vs. physical pain videos. Additionally, pain type × group interaction effect was significant, with a group difference more apparent for social pain *F*(1, 85) = 5.8, *p* = 0.018, ηp^2^ = 0.064.

##### Empathic arousal

The HNG scored significantly lower in the arousal component of affective empathy, *F* (1, 85) = 13.93, *p* < 0.001, ηp^2^ = 0.141 for both pain types. However, the main effect of pain type was not significant *F*(1, 85) = 2.46, *p* = 0.121 and the interaction between the pain type x group was insignificant, *F*(1, 85) = 3.54, *p* = 0.063.

##### Empathic concern

When asked about the person in the video, the LNG had significantly higher levels of empathic concern compared to the HNG, *F*(1, 85) = 9.75, *p* = 0.002, ηp^2^ = 0.103 in response to both types of pain. Furthermore, there was a significant main effect of pain type, *F*(1, 85) = 3.8, *p* = 0.054, ηp^2^ = 0.043, suggesting that participants in both groups reported greater empathic concern toward social pain than physical pain. The interaction of pain type × group was significant, with a group difference only apparent for social pain *F*(1, 85) = 5.68, *p* = 0.019, ηp^2^ = 0.063.

##### Empathic pain

**Figure 2:**
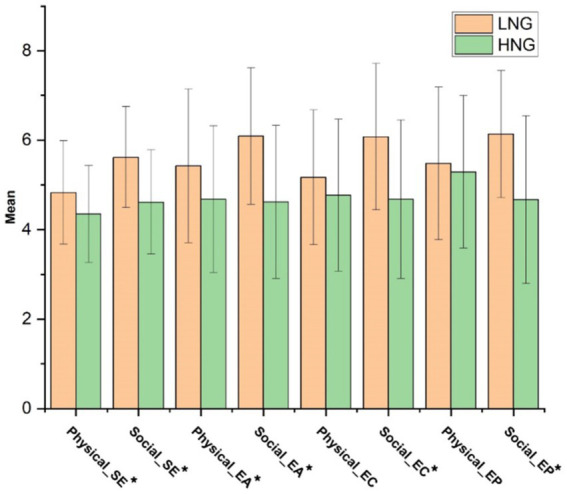
Mean of the components of affective empathy in two groups in response to physical and social pain. LNG, Low narcissistic group; HNG, High narcissistic group; SE, Specific emotions; EA, Empathic arousal; EC, Empathic concern; EP, Empathic pain. ^*^ indicates significant difference at *p* < 0.05.

**Figure 3:**
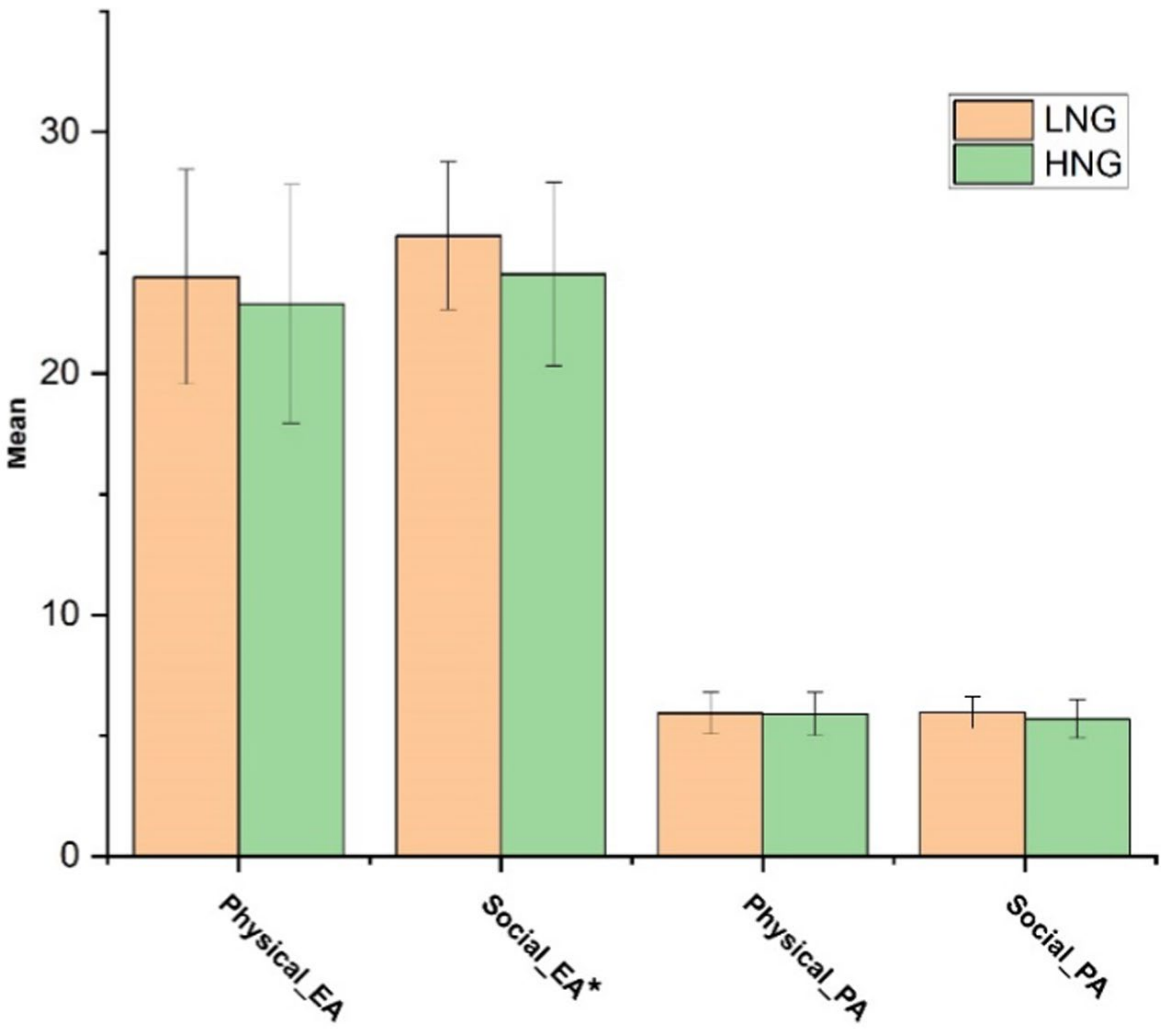
Mean of the components of empathic accuracy in two groups in response to physical and social pain. LNG, Low narcissistic group; HNG, High narcissistic group; EA, Emotion attribution; and PA, Pain attribution. ^*^ indicates significant difference at *p* < 0.05.

The HNG experienced significantly lower levels of pain than the LNG in response to both physical and social pain videos *F*(1, 85) = 7.62, *p* = 0.007, ηp^2^ = 0.082. In contrast, the main effect of pain type was insignificant within each group, *F*(1, 85) = 0.009, *p* = 0.926. Additionally, the pain type × group interaction was significant, with a group difference only apparent for social pain *F*(1, 85) = 9.98, *p* = 0.002, ηp^2^ = 0.105 (see Table 3 for descriptive statistics). Figures 2, 3 illustrate components of affective empathy and empathic accuracy in the HNG compared to the LNG in response to physical and social pain, respectively.

#### Self-other distinction measures

When comparing participants’ own emotional response to the videos, versus the emotions they rated for the target (the primary measure of self-other distinction), the HNG showed significantly greater disparity between these ratings *F*(1, 85) = 8.78, *p* = 0.004, ηp^2^ = 0.094. There was also a significant interaction between group and pain type, with a group difference only apparent for social pain *F*(1, 85) = 6.09, *p* = 0.016, ηp^2^ = 0.067. Although equivalent comparisons for pain did not reveal a significant main effect of group *F*(1, 85) = 1.24, *p* = 0.267, ηp^2^ = 0.014, and pain type *F*(1, 85) = 2.23, *p* = 0.139, ηp^2^ = 0.026, there was a significant interaction, with a greater disparity between ratings for the self and target for the HNG in relation to social pain videos only *F*(1, 85) = 9.23, *p* = 0.003, ηp^2^ = 0.098.

When comparing participants’ emotional reactions while watching the videos to the emotions targets felt when recording the videos, the LNG reported significantly greater emotion resonance with targets, *F*(1, 85) = 6.89, *p* = 0.010, ηp^2^ = 0.075. A significant interaction indicated a greater difference between groups for social pain only *F*(1, 85) = 6.12, *p* = 0.015, ηp^2^ = 0.067. Similar comparisons for pain, revealed the LNG also had significantly greater resonance with the targets in experiencing pain *F*(1, 85) = 7.81, *p* = 0.006, ηp^2^ = 0.084, with a significant interaction indicating a group difference only for social pain *F*(1, 85) = 9.5, *p* = 0.003, ηp^2^ = 0.101.

Finally, when considering participant arousal in response to videos and the target’s intensity ratings when recording the videos, the HNG again showed a significantly greater difference to targets versus the LNG *F*(1, 85) = 13.93, *p* = 0.000, ηp^2^ = 0.141. However the interaction between group and pain type was not significant *F*(1, 85) = 3.54, *p* = 0.063, ηp^2^ = 0.040 (see Table 3 for descriptive statistics).

As expected, the within-participant measure of self-other distinction was positively associated with the resonance measures, i.e., as the difference between the participants’ ratings for themselves and the target increased (primary measure) so did the difference between ratings taken from the participant and from the target (see Supplementary material 1).

**Figure 4:**
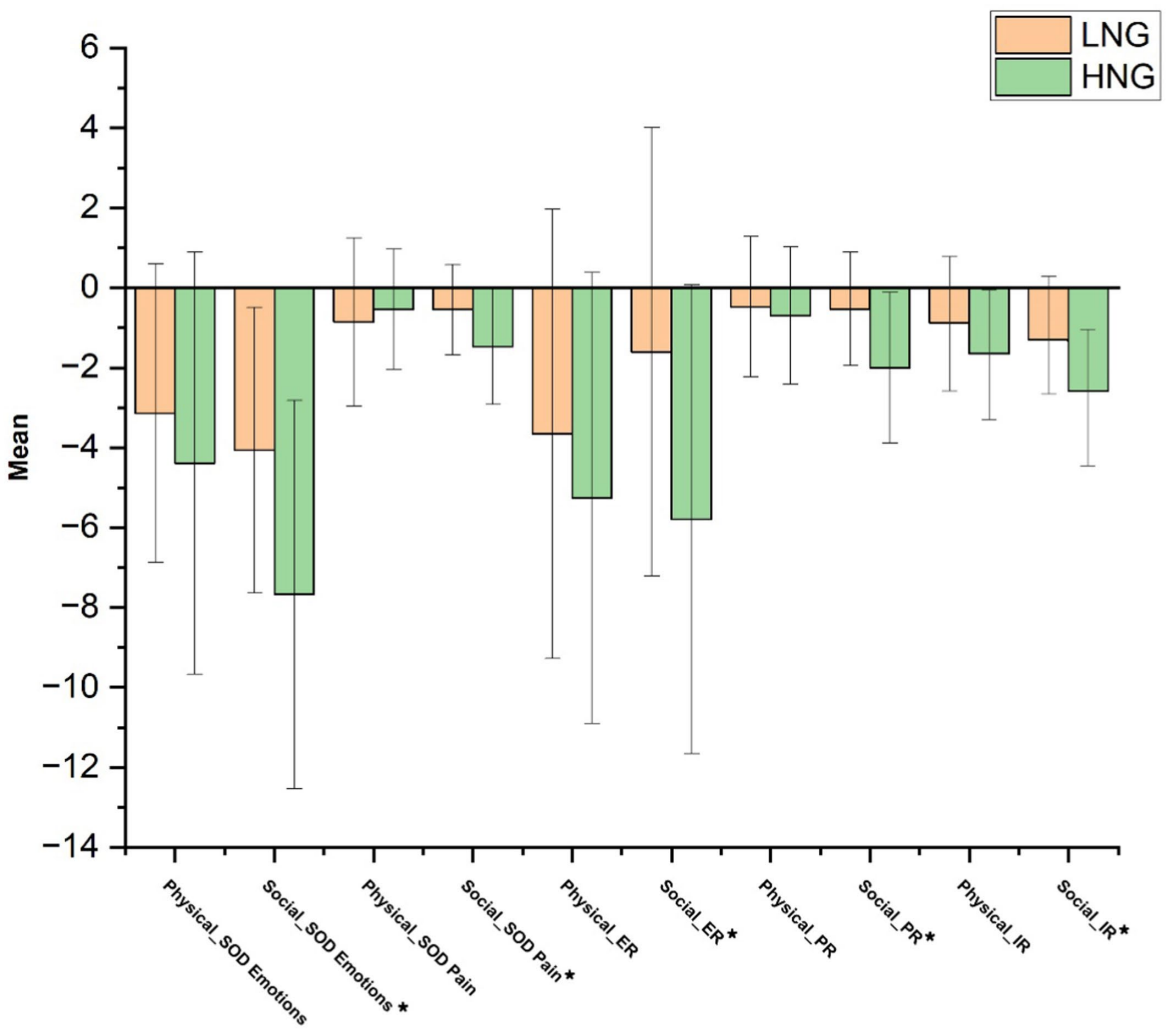
Mean of components related to self-other distinction for the high and low narcissism groups in response to physical and social pain. LNG, Low narcissistic group; HNG, High narcissistic group; ER, Emotional resonance; PR, Pain resonance; and IR, Intensity resonance. ^*^ indicates significant difference at *p* < 0.05.

In summary, both the primary measure of self-other distinction (within-participant measure) and resonance measures we expected to be closely linked to self-other distinction (between-participant comparisons) were suggestive of higher self-other distinction in the HNG for social pain. Figure 4 illustrates components of self-other distinction in the HNG compared to the LNG in response to physical and social pain, respectively.

#### Gender related analyses

Due to uneven gender distribution in our groups, we conducted further analyses with narcissism group and gender as between-subject factors, assessing their interactions across all dependent variables: subscales of empathic accuracy, affective empathy, and self-other distinction. These analyses did not reveal significant group × gender interactions, as indicated by the pairwise comparisons (see Supplementary material 2). In examining the interactions of gender x pain type, significant findings were limited to three variables: affective empathy—empathic concern [*F* (1, 84) = 5.95, *p* = 0.017, ηp^2^ = 0.066], and specific emotions [*F*(1, 84) = 3.93, *p* = 0.050, ηp^2^ = 0.045], and Self-other distinction—emotional resonance [*F*(1, 84) = 4.96, *p* = 0.029, ηp^2^ = 0.056], suggesting that women, overall and without considering the role of narcissism, tended to be more empathic and exhibited lower self-other distinction in response to the social pain of others. However, these interactions did not result in significant differences between genders within our groups; the significant differences were observed only across types of pain. Furthermore, the interaction of group × gender × pain type was not significant for any dependent variable. These results suggest gender does not significantly impact our primary outcomes (see Supplementary material 2).

## Discussion

In the current study, we used physical and social pain videos featuring targets expressing genuine emotion, in order to examine empathic accuracy, affective empathy, and differences in affective pain ratings for self and other for individuals with high and low levels of grandiose narcissism. Given our use of the NPI-16, moving forwards, our conclusions about narcissism are more likely to pertain to the grandiose subtype.

Our findings revealed that, when faced with either form of pain, individuals with high narcissistic traits displayed lower levels of affective empathy across all examined subcomponents (i.e., empathic concern, empathic arousal, empathic pain, and empathic emotions). The results remained consistent regardless of the measurement methods used, which included a video-task and the Interpersonal Reactivity Index (IRI). This finding aligns with recent studies including meta-analyses investigating the relationship between narcissism and affective empathy (Vonk et al., 2013; Chukwuorji et al., 2020; Urbonaviciute and Hepper, 2020; Eddy, 2021; Jacobs, 2022; Simard et al., 2022; di Giacomo et al., 2023).

In relation to cognitive empathy in narcissism, perhaps the picture is less clear. In our study, self-report data (IRI) revealed no difference between the high and low narcissistic groups in perspective-taking and fantasy, subcomponents of cognitive empathy. Yet, when we used the video-task method, individuals with high narcissistic traits showed a reduced ability in empathic accuracy in response to both types of pain, in terms of their attributions of degree of emotions felt by targets. This pattern is consistent with the findings of Pajevic et al., 2018, which showed a discrepancy between high self-reported cognitive empathy and actual performance in emotion recognition tasks. Such a difference could stem from a tendency among narcissists to overestimate their empathetic capabilities (Ronningstam, 2016; Bloxsom et al., 2021). Empathic accuracy (in terms of looking specifically at degree of specific emotions and pain) is an underexplored area. The current results are however consistent with Chukwuorji et al.’s (2020) finding of a negative correlation between all forms of empathy. Additionally, our study supports the idea that maladaptive aspects of narcissism, and perhaps those closely associated with grandiose narcissism, may hamper the accuracy of “mind reading” as suggested by emotion identification results (Hart et al., 2018). These disparities can be attributed not only to deficiencies but also to motivational factors in high narcissistic individuals (Baskin-Sommers et al., 2014; Hepper et al., 2014b; Lee and Kang, 2020), making it challenging to distinguish between the two.

Given the likely relationship between the ability to attribute emotions correctly to another person, and to feel and experience similar emotions in oneself, it is not unexpected to find narcissistic individuals exhibit deficits in both processes. However, when we consider measures more likely to reflect cognitive or affective empathy in the current study, we found more evidence for a significant issue with affective empathy. It is worth noting that narcissists still possess some level of empathic accuracy and what may distinguish them more clearly from people low in narcissism is their inner emotional resonance with others, or at least their self-awareness of any feelings that align with others’ emotional states.

Perceiving pain, or comprehending the level of pain another person is dealing with, was another rather novel focus of this study. Our findings suggest that people with narcissistic tendencies are adept at understanding both the physical and social pain that others experience. Essentially, it appears that whereas narcissistic individuals can correctly recognize others’ pain, they struggle to accurately attribute the degree of specific emotions linked to that pain. This may stem from the need for deeper engagement to comprehend another’s emotions rather than just their pain. The discrepancy may result from a reduced tendency to personally feel that pain and its related emotions in narcissism.

Another strength of the current study was that by measuring empathic accuracy in terms of the amount of emotions felt and perceived, we were able to calculate differences between self and other attributions. Our results showed that the HNG showed significantly greater differentiation between the pain and emotions attributed to targets in the pain videos and their own pain and emotions in response to observing those videos for social pain. This supported our initial hypothesis that more narcissistic people may show tendencies toward greater self-other distinction (Eddy, 2022). Greater self-other distinction aligns with diminished affective empathy, whereby highly narcissistic individuals may be less emotionally moved by others’ pain, and may focus more on themselves instead of empathizing with the distress of another person. This possibility is further supported by our other findings, including the lower arousal and emotional resonance in response to video targets expressing social pain, as well as lower empathic concern and personal distress in everyday life, as found in the HNG. However, because all of our measures were essentially self-report, it is not clear whether the lower ratings seen in the HNG truly reflect reduced ability to mirror the emotions of others, or whether these individuals were simply less attentive toward, accurate, or honest in relation to their affective responses to the videos. The fact that self-other distinction was only significantly greater for the HNG in relation to social pain is worthy of further exploration, but it may reflect the socially competitive nature of many narcissistic traits. High self-other distinction may characterize many social cognitive strategies used by individuals with narcissism to maintain their status and/or emotional equilibrium, and is perhaps more likely to predict low affective empathy than impairments in mentalising or cognitive empathy (Eddy, 2021, 2022).

It is notable that our findings indicated that both the HNG and LNG displayed greater empathic accuracy in response to videos depicting social pain as compared to those showing physical pain. This response reflects our shared sensitivity toward social pain, a common part of our everyday lives. Despite their accuracy in understanding others’ emotions during social pain, narcissists felt fewer empathic arousal, empathic concern, and empathic pain in these situations compared to those involving physical pain. This makes it all more interesting that self-other distinction was higher for social pain. It seems that for highly narcissistic individuals, the more adept they are at recognizing others’ social pain compared to physical pain, the less they empathize with them emotionally. This confirms our hypothesis that individuals with high narcissism traits disengaged more with the social pain of others compared to their physical pain. One explanation for this is that narcissists, despite their heightened sensitivity to social pain, suppress it more vehemently when witnessing it in others, and exhibit higher levels of self-other distinction to avoid experiencing a similar discomfort—an emotion they find highly undesirable. In other words, there is likely to be a motivational effect, e.g., a defensive mechanism intended to protect their ego. The second interpretation suggests that as grandiose narcissists remain relatively unaffected when facing social pain stimuli, often externalizing it as someone else’s problem, not theirs (Brunell et al., 2021), and so they find it challenging to empathize when others are going through such experiences. In other words, highly narcissistic individuals may either choose to detach from, or just be rather emotionally insensitive to, other’s affective states. Perhaps this may be more likely in the context of social pain as this is a more personally disturbing experience, whereas physical pain, which is a potential threat to themselves as well, draws their attention and engages them more effectively.

Understanding the social pain of others without feeling it may help to explain grandiose narcissistic characteristics such as the exploitation of others (Lamm et al., 2016; Di Pierro et al., 2018; di Giacomo et al., 2023; Duradoni et al., 2023), and the volatility often seen in narcissistic romantic relationships, where social pain as a result of rejection and abandonment (e.g., the “silent treatment”), is highly relevant, and may even be applied instrumentally by highly narcissistic individuals (Eddy, 2021).

Our study encountered certain limitations, notably our focus on the grandiose subtype of narcissism. Future research should also explore the vulnerable subtype and other conceptualizations of narcissism, such as the three-factor model (agentic extraversion, narcissistic neuroticism, and antagonism/entitlement; Simard et al., 2022), to provide a more comprehensive understanding. The second limitation pertains to the uneven distribution of gender within our sample, which can be attributed to the initial recruitment process and reflects the presence of narcissism within the sample population. Although our sample was in line with previous research suggesting that men tend to exhibit higher levels of narcissism compared to women (Grijalva et al., 2015; Chukwuorji et al., 2020), it is important to note that this imbalance in gender representation may influence the applicability of our findings to other populations. Another limitation of our study is the absence of control videos presenting neutral or positive situations, which could offer a comparison point to pain-related scenarios, similar to the methods employed by Atkins et al., 2016 and Riva et al., 2011. Our findings indicated that the baseline mood state of individuals with high narcissistic traits was more upbeat than those with low traits, aligning with previous research (Sedikides et al., 2004; Zuckerman and O'Loughlin, 2009). However, both groups displayed similar degrees of negative affect, which likely did not influence their empathic responses to others’ pain. Given that pain is typically unassociated with positive emotion and negative emotion plays a more significant role in dysfunctional interactions often linked with narcissism, this aspect was a focal point of our study. Nevertheless, it is important to acknowledge that the persistent positive mood observed in narcissistic individuals during their daily lives, introduces an uncontrollable element that could potentially influence their empathic responses in everyday situations. Therefore, exploring their responses to neutral and positive stimuli is a crucial area for future research, offering insight into the full spectrum of empathic responses in narcissistic individuals. In addition, it may be beneficial for future research to include a neutral group of referees to offer a more objective assessment of target expressivity. Studies could also explore whether self-other distinction varies according to narcissistic subtype, or mediates the association between narcissism and empathy, topics beyond the scope of our current study but which hold significant promise for advancing our understanding. Employing the validated, longer version of the Interpersonal Reactivity Index (IRI) in similar studies is also advisable. Furthermore, given that the current sample was limited to a healthy university population, future research should aim to replicate our findings in clinical samples among individuals diagnosed with Narcissistic Personality Disorder. Finally, we acknowledge that current conceptualizations of self-other distinction require refinement, and that there is no current consensus as to the best measure of this construct. Indeed, self-other distinction may be difficult to measure given that it could be intrinsically, and perhaps even differentially, linked to specific kinds of mental or physical states. An individual may say that they feel they are completely different from someone else, but at the same time, they may actually show evidence of significant resonance with another person, which may seem to suggest low self-other distinction. We hoped to help encompass this possibility by including both a within-participant measure of self-other distinction, and the resonance measures in the current study (which may be considered to represent explicit, and implicit measures of self-other distinction, respectively). At the same time, we accept the challenges associated with investigating such a complex construct, and the potential limitations associated with both intra- and inter-individual measures of self-other distinction.

## Conclusion

In conclusion, highly narcissistic individuals can show lower affective empathy in response to observation of others’ social pain, and their attributions of the degree of emotion felt by those others may also be less accurate than the attributions made by individuals low in narcissism. We report preliminary evidence that grandiose narcissistic traits may also predict increased discrepancy between emotion ratings for self vs. other in response to observing those others’ social pain, in addition to reduced emotional resonance, personal distress and empathic concern, supporting the likelihood of higher self-other distinction in more narcissistic individuals.

## Data availability statement

The datasets presented in this study can be found in online repositories. The names of the repository/repositories and accession number(s) can be found at: https://doi.org/10.6084/m9.figshare.23937063.

## Ethics statement

The studies involving humans were approved by Shahid Beheshti University (IR.SBU.REC.1402.044). The studies were conducted in accordance with the local legislation and institutional requirements. The participants provided their written informed consent to participate in this study.

## Author contributions

FS: Conceptualization, Formal Analysis, Methodology, Investigation, Visualization, Writing – original draft. AZ: Conceptualization, Methodology, Supervision, Writing – review & editing. AT: Conceptualization, Methodology, Formal Analysis, Writing – review & editing. MEH: Formal Analysis, Software, Investigation, Visualization, Writing – review & editing. MR: Resources, Writing – review & editing. AS: Resources, Writing – review & editing. CE: Conceptualization, Methodology, Writing – original draft.

## Supplementary material

Supplementary Material 1: Self-other Distinction Measures.

Supplementary Material 2: Gender-Related Analyses.
