# Supplementary material for: I understand your pain but I do not feel it: lower affective empathy in response to others’ social pain in narcissism
Source: Front Psychol. 2024 Mar 15;15:1350133. doi: 10.3389/fpsyg.2024.1350133 (PMC10994002; doi:10.3389/fpsyg.2024.1350133)
Supplement: Supplementary Material 1: — Self-other Distinction Measures. [file Data_Sheet_1.docx]

Pain_Type1 =Social Pain, Pain_Type2 =Physical Pain

Group1=LNG (Low Narcissistic Group), Group2=HNG (High Narcissistic Group)

**Empathic Accuracy**

Dependent Variable 🡪 Emotion Attribution

| Source | | df | *F* | *p* | Partial Eta Squared | Observed Power^a^ |
| --- | --- | --- | --- | --- | --- | --- |
| Pain_Type | Sphericity Assumed | 1 | 3.995 | .049 | .045 | .506 |
|  | Greenhouse-Geisser | 1.000 | 3.995 | .049 | .045 | .506 |
| Pain_Type * Gender | Sphericity Assumed | 1 | 2.086 | .152 | .024 | .298 |
|  | Greenhouse-Geisser | 1.000 | 2.086 | .152 | .024 | .298 |
| Pain_Type * group | Sphericity Assumed | 1 | .026 | .872 | .000 | .053 |
|  | Greenhouse-Geisser | 1.000 | .026 | .872 | .000 | .053 |
| Error(Pain_Type) | Sphericity Assumed | 84 |  |  |  |  |
|  | Greenhouse-Geisser | 84.000 |  |  |  |  |

| **Tests of Between-Subjects Effects** | | | | | | | | | |
| --- | --- | --- | --- | --- | --- | --- | --- | --- | --- |
| Transformed Variable: Average | | | | | | | | | |
| Source | Type III Sum of Squares | df | Mean Square | F | Sig. | Partial Eta Squared | Noncent. Parameter | Observed Power^a^ |  |
| Intercept | 206623.519 | 1 | 206623.519 | 26826.872 | .000 | .997 | 26826.872 | 1.000 |  |
| Gender | 1.613 | 1 | 1.613 | .209 | .648 | .002 | .209 | .074 |  |
| group | 33.206 | 1 | 33.206 | 4.311 | .041 | .049 | 4.311 | .537 |  |
| Error | 646.977 | 84 | 7.702 |  |  |  |  |  |  |
| a. Computed using alpha = .05 | | | | | | | | | |

| **Pairwise Comparisons** | | | | | | | | |
| --- | --- | --- | --- | --- | --- | --- | --- | --- |
| Measure: MEASURE_1 | | | | | | | | |
| group | Pain_Type | (I) Gender | (J) Gender | Mean Difference (I-J) | Std. Error | Sig.^a^ | 95% Confidence Interval for Difference^a^ | |
|  |  |  |  |  |  |  | Lower Bound | Upper Bound |
| 1 | 1 | Female | Male | .354 | .509 | .489 | -.659 | 1.368 |
|  |  | Male | Female | -.354 | .509 | .489 | -1.368 | .659 |
|  | 2 | Female | Male | -.767 | .669 | .255 | -2.098 | .564 |
|  |  | Male | Female | .767 | .669 | .255 | -.564 | 2.098 |
| 2 | 1 | Female | Male | .354 | .509 | .489 | -.659 | 1.368 |
|  |  | Male | Female | -.354 | .509 | .489 | -1.368 | .659 |
|  | 2 | Female | Male | -.767 | .669 | .255 | -2.098 | .564 |
|  |  | Male | Female | .767 | .669 | .255 | -.564 | 2.098 |
| Based on estimated marginal means | | | | | | | | |
| a. Adjustment for multiple comparisons: Bonferroni. | | | | | | | | |

| **Pairwise Comparisons** | | | | | | | |
| --- | --- | --- | --- | --- | --- | --- | --- |
| group | (I) Gender | (J) Gender | Mean Difference (I-J) | Std. Error | Sig.^a^ | 95% Confidence Interval for Difference^a^ | |
|  |  |  |  |  |  | Lower Bound | Upper Bound |
| 1 | Female | Male | -.206 | .451 | .648 | -1.103 | .690 |
|  | Male | Female | .206 | .451 | .648 | -.690 | 1.103 |
| 2 | Female | Male | -.206 | .451 | .648 | -1.103 | .690 |
|  | Male | Female | .206 | .451 | .648 | -.690 | 1.103 |
| Based on estimated marginal means | | | | | | | |
| a. Adjustment for multiple comparisons: Bonferroni. | | | | | | | |
| Group1= LNG, Group2= HNG | | | | | | | |

Dv 🡪 Pain Attribution

| Source | | df | F | p | Partial Eta Squared | Observed Power^a^ |
| --- | --- | --- | --- | --- | --- | --- |
| Pain_Type | Sphericity Assumed | 1 | .651 | .422 | .008 | .125 |
|  | Greenhouse-Geisser | 1.000 | .651 | .422 | .008 | .125 |
| Pain_Type * Gender | Sphericity Assumed | 1 | .387 | .535 | .005 | .094 |
|  | Greenhouse-Geisser | 1.000 | .387 | .535 | .005 | .094 |
| Pain_Type * group | Sphericity Assumed | 1 | .591 | .444 | .007 | .118 |
|  | Greenhouse-Geisser | 1.000 | .591 | .444 | .007 | .118 |
| Error(Pain_Type) | Sphericity Assumed | 84 |  |  |  |  |
|  | Greenhouse-Geisser | 84.000 |  |  |  |  |

| **Tests of Between-Subjects Effects** | | | | | | | | |
| --- | --- | --- | --- | --- | --- | --- | --- | --- |
| Transformed Variable: Average | | | | | | | | |
| Source | Type III Sum of Squares | df | Mean Square | F | Sig. | Partial Eta Squared | Noncent. Parameter | Observed Power^a^ |
| Intercept | 5992.039 | 1 | 5992.039 | 8061.631 | .000 | .990 | 8061.631 | 1.000 |
| Gender | 2.547 | 1 | 2.547 | 3.427 | .068 | .039 | 3.427 | .448 |
| group | 2.307 | 1 | 2.307 | 3.104 | .082 | .036 | 3.104 | .414 |
| Error | 62.435 | 84 | .743 |  |  |  |  |  |
| a. Computed using alpha = .05 | | | | | | | | |

| **Pairwise Comparisons** | | | | | | | | | |
| --- | --- | --- | --- | --- | --- | --- | --- | --- | --- |
| Measure: MEASURE_1 | | | | | | | | | |
| group | Pain_Type | (I) Gender | (J) Gender | Mean Difference (I-J) | Std. Error | Sig.^a^ | 95% Confidence Interval for Difference^a^ | |  |
|  |  |  |  |  |  |  | Lower Bound | Upper Bound |  |
| 1 | 1 | Female | Male | -.185 | .168 | .273 | -.519 | .148 |  |
|  |  | Male | Female | .185 | .168 | .273 | -.148 | .519 |  |
|  | 2 | Female | Male | -.333 | .198 | .097 | -.728 | .061 |  |
|  |  | Male | Female | .333 | .198 | .097 | -.061 | .728 |  |
| 2 | 1 | Female | Male | -.185 | .168 | .273 | -.519 | .148 |  |
|  |  | Male | Female | .185 | .168 | .273 | -.148 | .519 |  |
|  | 2 | Female | Male | -.333 | .198 | .097 | -.728 | .061 |  |
|  |  | Male | Female | .333 | .198 | .097 | -.061 | .728 |  |
| Based on estimated marginal means | | | | | | | | | |
| a. Adjustment for multiple comparisons: Bonferroni. | | | | | | | | | |

| **Pairwise Comparisons** | | | | | | | |
| --- | --- | --- | --- | --- | --- | --- | --- |
| group | (I) Gender | (J) Gender | Mean Difference (I-J) | Std. Error | Sig.^a^ | 95% Confidence Interval for Difference^a^ | |
|  |  |  |  |  |  | Lower Bound | Upper Bound |
| 1 | Female | Male | -.259 | .140 | .068 | -.538 | .019 |
|  | Male | Female | .259 | .140 | .068 | -.019 | .538 |
| 2 | Female | Male | -.259 | .140 | .068 | -.538 | .019 |
|  | Male | Female | .259 | .140 | .068 | -.019 | .538 |
| Based on estimated marginal means | | | | | | | |
| a. Adjustment for multiple comparisons: Bonferroni. | | | | | | | |

Group1= LNG, Group2= HNG

**Affective Empathy**

Dv🡪 Specific Emotions

| Source | | df | F | p | Partial Eta Squared | Observed Power^a^ |
| --- | --- | --- | --- | --- | --- | --- |
| Pain_Type | Sphericity Assumed | 1 | 23.827 | .000 | .221 | .998 |
|  | Greenhouse-Geisser | 1.000 | 23.827 | .000 | .221 | .998 |
| Pain_Type * Gender | Sphericity Assumed | 1 | 3.939 | .050 | .045 | .501 |
|  | Greenhouse-Geisser | 1.000 | 3.939 | .050 | .045 | .501 |
| Pain_Type * group | Sphericity Assumed | 1 | 2.501 | .118 | .029 | .346 |
|  | Greenhouse-Geisser | 1.000 | 2.501 | .118 | .029 | .346 |
| Error(Pain_Type) | Sphericity Assumed | 84 |  |  |  |  |
|  | Greenhouse-Geisser | 84.000 |  |  |  |  |

| **Tests of Between-Subjects Effects** | | | | | | | | | | | | | | | | |
| --- | --- | --- | --- | --- | --- | --- | --- | --- | --- | --- | --- | --- | --- | --- | --- | --- |
|  | | | | | | | | | | | | | | | | |
| Transformed Variable: Average | | | | | | | | | | | | | | | | |
| Source | | Type III Sum of Squares | | df | | Mean Square | | F | Sig. | | Partial Eta Squared | | | Noncent. Parameter | | Observed Power^a^ |
| Intercept | | 4088.401 | | 1 | | 4088.401 | | 1965.895 | .000 | | .959 | | | 1965.895 | | 1.000 |
| Gender | | 1.206 | | 1 | | 1.206 | | .580 | .448 | | .007 | | | .580 | | .117 |
| group | | 17.478 | | 1 | | 17.478 | | 8.404 | .005 | | .091 | | | 8.404 | | .817 |
| Error | | 174.692 | | 84 | | 2.080 | |  |  | |  | | |  | |  |
| a. Computed using alpha = .05 | | | | | | | | | | | | | | | | |
| **Pairwise Comparisons** | | | | | | | | | | | | | | | | |
|  | | | | | | | | | | | | | | | | |
| group | Pain_Type | | (I) Gender | | (J) Gender | | Mean Difference (I-J) | | | Std. Error | | Sig.^a^ | 95% Confidence Interval for Difference^a^ | | | |
|  |  |  |  |  |  |  |  |  |  |  |  |  | Lower Bound | | Upper Bound | |
| 1 | 1 | | Female | | Male | | .405 | | | .262 | | .126 | -.116 | | .926 | |
|  |  |  | Male | | Female | | -.405 | | | .262 | | .126 | -.926 | | .116 | |
|  | 2 | | Female | | Male | | -.048 | | | .259 | | .853 | -.563 | | .467 | |
|  |  |  | Male | | Female | | .048 | | | .259 | | .853 | -.467 | | .563 | |
| 2 | 1 | | Female | | Male | | .405 | | | .262 | | .126 | -.116 | | .926 | |
|  |  |  | Male | | Female | | -.405 | | | .262 | | .126 | -.926 | | .116 | |
|  | 2 | | Female | | Male | | -.048 | | | .259 | | .853 | -.563 | | .467 | |
|  |  |  | Male | | Female | | .048 | | | .259 | | .853 | -.467 | | .563 | |
| Based on estimated marginal means | | | | | | | | | | | | | | | | |
| a. Adjustment for multiple comparisons: Bonferroni. | | | | | | | | | | | | | | | | |

| **Pairwise Comparisons** | | | | | | | |
| --- | --- | --- | --- | --- | --- | --- | --- |
| group | (I) Gender | (J) Gender | Mean Difference (I-J) | Std. Error | Sig.^a^ | 95% Confidence Interval for Difference^a^ | |
|  |  |  |  |  |  | Lower Bound | Upper Bound |
| 1 | Female | Male | .178 | .234 | .448 | -.287 | .644 |
|  | Male | Female | -.178 | .234 | .448 | -.644 | .287 |
| 2 | Female | Male | .178 | .234 | .448 | -.287 | .644 |
|  | Male | Female | -.178 | .234 | .448 | -.644 | .287 |
| Based on estimated marginal means | | | | | | | |
| 1. Adjustment for multiple comparisons: Bonferroni. | | | | | | | |

DV 🡪 Empathic Arousal

| Source | | df | F | Sig. | Partial Eta Squared | Observed Power^a^ |
| --- | --- | --- | --- | --- | --- | --- |
| Pain_Type | Sphericity Assumed | 1 | 2.309 | .132 | .027 | .324 |
|  | Greenhouse-Geisser | 1.000 | 2.309 | .132 | .027 | .324 |
| Pain_Type * Gender | Sphericity Assumed | 1 | 1.605 | .209 | .019 | .240 |
|  | Greenhouse-Geisser | 1.000 | 1.605 | .209 | .019 | .240 |
| Pain_Type * group | Sphericity Assumed | 1 | 1.719 | .193 | .020 | .254 |
|  | Greenhouse-Geisser | 1.000 | 1.719 | .193 | .020 | .254 |
| Error(Pain_Type) | Sphericity Assumed | 84 |  |  |  |  |
|  | Greenhouse-Geisser | 84.000 |  |  |  |  |

| **Tests of Between-Subjects Effects** | | | | | | | | |
| --- | --- | --- | --- | --- | --- | --- | --- | --- |
|  | | | | | | | | |
| Transformed Variable: Average | | | | | | | | |
| Source | Type III Sum of Squares | df | Mean Square | F | Sig. | Partial Eta Squared | Noncent. Parameter | Observed Power^a^ |
| Intercept | 4700.427 | 1 | 4700.427 | 1215.724 | .000 | .935 | 1215.724 | 1.000 |
| Gender | 1.128 | 1 | 1.128 | .292 | .591 | .003 | .292 | .083 |
| group | 41.575 | 1 | 41.575 | 10.753 | .002 | .113 | 10.753 | .900 |
| Error | 324.774 | 84 | 3.866 |  |  |  |  |  |
| a. Computed using alpha = .05 | | | | | | | | |

| **Pairwise Comparisons** | | | | | | | | |
| --- | --- | --- | --- | --- | --- | --- | --- | --- |
|  | | | | | | | | |
| group | Pain_Type | (I) Gender | (J) Gender | Mean Difference (I-J) | Std. Error | Sig.^a^ | 95% Confidence Interval for Difference^a^ | |
|  |  |  |  |  |  |  | Lower Bound | Upper Bound |
| 1 | 1 | Female | Male | .434 | .372 | .247 | -.306 | 1.174 |
|  |  | Male | Female | -.434 | .372 | .247 | -1.174 | .306 |
|  | 2 | Female | Male | -.089 | .388 | .819 | -.861 | .683 |
|  |  | Male | Female | .089 | .388 | .819 | -.683 | .861 |
| 2 | 1 | Female | Male | .434 | .372 | .247 | -.306 | 1.174 |
|  |  | Male | Female | -.434 | .372 | .247 | -1.174 | .306 |
|  | 2 | Female | Male | -.089 | .388 | .819 | -.861 | .683 |
|  |  | Male | Female | .089 | .388 | .819 | -.683 | .861 |
| Based on estimated marginal means | | | | | | | | |
| a. Adjustment for multiple comparisons: Bonferroni. | | | | | | | | |

| **Pairwise Comparisons** | | | | | | | |
| --- | --- | --- | --- | --- | --- | --- | --- |
| group | (I) Gender | (J) Gender | Mean Difference (I-J) | Std. Error | Sig.^a^ | 95% Confidence Interval for Difference^a^ | |
|  |  |  |  |  |  | Lower Bound | Upper Bound |
| 1 | Female | Male | .172 | .319 | .591 | -.463 | .808 |
|  | Male | Female | -.172 | .319 | .591 | -.808 | .463 |
| 2 | Female | Male | .172 | .319 | .591 | -.463 | .808 |
|  | Male | Female | -.172 | .319 | .591 | -.808 | .463 |
| Based on estimated marginal means | | | | | | | |
| a. Adjustment for multiple comparisons: Bonferroni. | | | | | | | |

DV 🡪 Empathic Concern

| Source | | df | F | p | Partial Eta Squared | Observed Power^a^ |
| --- | --- | --- | --- | --- | --- | --- |
| Pain_Type | Sphericity Assumed | 1 | 3.624 | .060 | .041 | .469 |
|  | Greenhouse-Geisser | 1.000 | 3.624 | .060 | .041 | .469 |
| Pain_Type * Gender | Sphericity Assumed | 1 | 5.955 | .017 | .066 | .674 |
|  | Greenhouse-Geisser | 1.000 | 5.955 | .017 | .066 | .674 |
| Pain_Type * group | Sphericity Assumed | 1 | 2.018 | .159 | .023 | .290 |
|  | Greenhouse-Geisser | 1.000 | 2.018 | .159 | .023 | .290 |
| Error(Pain_Type) | Sphericity Assumed | 84 |  |  |  |  |
|  | Greenhouse-Geisser | 84.000 |  |  |  |  |

| **Tests of Between-Subjects Effects** | | | | | | | | |
| --- | --- | --- | --- | --- | --- | --- | --- | --- |
|  | | | | | | | | |
| Transformed Variable: Average | | | | | | | | |
| Source | Type III Sum of Squares | df | Mean Square | F | Sig. | Partial Eta Squared | Noncent. Parameter | Observed Power^a^ |
| Intercept | 4647.496 | 1 | 4647.496 | 1283.124 | .000 | .939 | 1283.124 | 1.000 |
| Gender | .771 | 1 | .771 | .213 | .646 | .003 | .213 | .074 |
| group | 27.155 | 1 | 27.155 | 7.497 | .008 | .082 | 7.497 | .772 |
| Error | 304.249 | 84 | 3.622 |  |  |  |  |  |
| a. Computed using alpha = .05 | | | | | | | | |

| **Pairwise Comparisons** | | | | | | | | |
| --- | --- | --- | --- | --- | --- | --- | --- | --- |
|  | | | | | | | | |
| group | Pain_Type | (I) Gender | (J) Gender | Mean Difference (I-J) | Std. Error | Sig.^a^ | 95% Confidence Interval for Difference^a^ | |
|  |  |  |  |  |  |  | Lower Bound | Upper Bound |
| 1 | 1 | Female | Male | .674 | .387 | .085 | -.096 | 1.444 |
|  |  | Male | Female | -.674 | .387 | .085 | -1.444 | .096 |
|  | 2 | Female | Male | -.389 | .369 | .295 | -1.123 | .345 |
|  |  | Male | Female | .389 | .369 | .295 | -.345 | 1.123 |
| 2 | 1 | Female | Male | .674 | .387 | .085 | -.096 | 1.444 |
|  |  | Male | Female | -.674 | .387 | .085 | -1.444 | .096 |
|  | 2 | Female | Male | -.389 | .369 | .295 | -1.123 | .345 |
|  |  | Male | Female | .389 | .369 | .295 | -.345 | 1.123 |
| Based on estimated marginal means | | | | | | | | |
| a. Adjustment for multiple comparisons: Bonferroni. | | | | | | | | |

| **Pairwise Comparisons** | | | | | | | |
| --- | --- | --- | --- | --- | --- | --- | --- |
| group | (I) Gender | (J) Gender | Mean Difference (I-J) | Std. Error | Sig.^a^ | 95% Confidence Interval for Difference^a^ | |
|  |  |  |  |  |  | Lower Bound | Upper Bound |
| 1 | Female | Male | .143 | .309 | .646 | -.472 | .757 |
|  | Male | Female | -.143 | .309 | .646 | -.757 | .472 |
| 2 | Female | Male | .143 | .309 | .646 | -.472 | .757 |
|  | Male | Female | -.143 | .309 | .646 | -.757 | .472 |
| Based on estimated marginal means | | | | | | | |
| a. Adjustment for multiple comparisons: Bonferroni. | | | | | | | |

DV 🡪 Empathic Pain

| Source | | df | F | Sig. | Partial Eta Squared | Observed Power^a^ |
| --- | --- | --- | --- | --- | --- | --- |
| Pain_Type | Sphericity Assumed | 1 | .001 | .969 | .000 | .050 |
|  | Greenhouse-Geisser | 1.000 | .001 | .969 | .000 | .050 |
| Pain_Type * Gender | Sphericity Assumed | 1 | 1.766 | .188 | .021 | .260 |
|  | Greenhouse-Geisser | 1.000 | 1.766 | .188 | .021 | .260 |
| Pain_Type * group | Sphericity Assumed | 1 | 6.199 | .015 | .069 | .692 |
|  | Greenhouse-Geisser | 1.000 | 6.199 | .015 | .069 | .692 |
| Error(Pain_Type) | Sphericity Assumed | 84 |  |  |  |  |
|  | Greenhouse-Geisser | 84.000 |  |  |  |  |

| **Tests of Between-Subjects Effects** | | | | | | | | |
| --- | --- | --- | --- | --- | --- | --- | --- | --- |
|  | | | | | | | | |
| Transformed Variable: Average | | | | | | | | |
| Source | Type III Sum of Squares | df | Mean Square | F | Sig. | Partial Eta Squared | Noncent. Parameter | Observed Power^a^ |
| Intercept | 5047.828 | 1 | 5047.828 | 1299.246 | .000 | .939 | 1299.246 | 1.000 |
| Gender | 4.132 | 1 | 4.132 | 1.064 | .305 | .013 | 1.064 | .175 |
| group | 18.994 | 1 | 18.994 | 4.889 | .030 | .055 | 4.889 | .589 |
| Error | 326.357 | 84 | 3.885 |  |  |  |  |  |
| a. Computed using alpha = .05 | | | | | | | | |

| **Pairwise Comparisons** | | | | | | | | |
| --- | --- | --- | --- | --- | --- | --- | --- | --- |
|  | | | | | | | | |
| group | Pain_Type | (I) Gender | (J) Gender | Mean Difference (I-J) | Std. Error | Sig.^a^ | 95% Confidence Interval for Difference^a^ | |
|  |  |  |  |  |  |  | Lower Bound | Upper Bound |
| 1 | 1 | Female | Male | .617 | .379 | .107 | -.136 | 1.370 |
|  |  | Male | Female | -.617 | .379 | .107 | -1.370 | .136 |
|  | 2 | Female | Male | .043 | .394 | .913 | -.740 | .826 |
|  |  | Male | Female | -.043 | .394 | .913 | -.826 | .740 |
| 2 | 1 | Female | Male | .617 | .379 | .107 | -.136 | 1.370 |
|  |  | Male | Female | -.617 | .379 | .107 | -1.370 | .136 |
|  | 2 | Female | Male | .043 | .394 | .913 | -.740 | .826 |
|  |  | Male | Female | -.043 | .394 | .913 | -.826 | .740 |
| Based on estimated marginal means | | | | | | | | |
| a. Adjustment for multiple comparisons: Bonferroni. | | | | | | | | |

| **Pairwise Comparisons** | | | | | | | |
| --- | --- | --- | --- | --- | --- | --- | --- |
| group | (I) Gender | (J) Gender | Mean Difference (I-J) | Std. Error | Sig.^a^ | 95% Confidence Interval for Difference^a^ | |
|  |  |  |  |  |  | Lower Bound | Upper Bound |
| 1 | Female | Male | .330 | .320 | .305 | -.306 | .967 |
|  | Male | Female | -.330 | .320 | .305 | -.967 | .306 |
| 2 | Female | Male | .330 | .320 | .305 | -.306 | .967 |
|  | Male | Female | -.330 | .320 | .305 | -.967 | .306 |
| Based on estimated marginal means | | | | | | | |
| a. Adjustment for multiple comparisons: Bonferroni. | | | | | | | |

**Self-Other Distinction**

DV 🡪 Intensity Resonanse

| **Tests of Within-Subjects Effects** | | | | | | | | | |
| --- | --- | --- | --- | --- | --- | --- | --- | --- | --- |
| Source | | Type III Sum of Squares | df | Mean Square | F | Sig. | Partial Eta Squared | Noncent. Parameter | Observed Power^a^ |
| Pain_Type | Sphericity Assumed | 40.583 | 1 | 40.583 | 2.359 | .128 | .028 | 2.359 | .330 |
|  | Greenhouse-Geisser | 40.583 | 1.000 | 40.583 | 2.359 | .128 | .028 | 2.359 | .330 |
|  | Huynh-Feldt | 40.583 | 1.000 | 40.583 | 2.359 | .128 | .028 | 2.359 | .330 |
|  | Lower-bound | 40.583 | 1.000 | 40.583 | 2.359 | .128 | .028 | 2.359 | .330 |
| Pain_Type * group | Sphericity Assumed | 1.056 | 1 | 1.056 | .061 | .805 | .001 | .061 | .057 |
|  | Greenhouse-Geisser | 1.056 | 1.000 | 1.056 | .061 | .805 | .001 | .061 | .057 |
|  | Huynh-Feldt | 1.056 | 1.000 | 1.056 | .061 | .805 | .001 | .061 | .057 |
|  | Lower-bound | 1.056 | 1.000 | 1.056 | .061 | .805 | .001 | .061 | .057 |
| Pain_Type * Gender | Sphericity Assumed | 6.933 | 1 | 6.933 | .403 | .527 | .005 | .403 | .096 |
|  | Greenhouse-Geisser | 6.933 | 1.000 | 6.933 | .403 | .527 | .005 | .403 | .096 |
|  | Huynh-Feldt | 6.933 | 1.000 | 6.933 | .403 | .527 | .005 | .403 | .096 |
|  | Lower-bound | 6.933 | 1.000 | 6.933 | .403 | .527 | .005 | .403 | .096 |
| Error(Pain_Type) | Sphericity Assumed | 1410.554 | 82 | 17.202 |  |  |  |  |  |
|  | Greenhouse-Geisser | 1410.554 | 82.000 | 17.202 |  |  |  |  |  |
|  | Huynh-Feldt | 1410.554 | 82.000 | 17.202 |  |  |  |  |  |
|  | Lower-bound | 1410.554 | 82.000 | 17.202 |  |  |  |  |  |
| a. Computed using alpha = .05 | | | | | | | | | |

| **Tests of Between-Subjects Effects** | | | | | | | | |
| --- | --- | --- | --- | --- | --- | --- | --- | --- |
|  | | | | | | | | |
| Transformed Variable: Average | | | | | | | | |
| Source | Type III Sum of Squares | df | Mean Square | F | Sig. | Partial Eta Squared | Noncent. Parameter | Observed Power^a^ |
| Intercept | 2260.853 | 1 | 2260.853 | 47.668 | .000 | .368 | 47.668 | 1.000 |
| group | 186.539 | 1 | 186.539 | 3.933 | .051 | .046 | 3.933 | .500 |
| Gender | 32.597 | 1 | 32.597 | .687 | .409 | .008 | .687 | .130 |
| Error | 3889.169 | 82 | 47.429 |  |  |  |  |  |
| a. Computed using alpha = .05 | | | | | | | | |

| **Pairwise Comparisons** | | | | | | | | |
| --- | --- | --- | --- | --- | --- | --- | --- | --- |
| Pain_Type | group | (I) Gender | (J) Gender | Mean Difference (I-J) | Std. Error | Sig.^a^ | 95% Confidence Interval for Difference^a^ | |
|  |  |  |  |  |  |  | Lower Bound | Upper Bound |
| 1 | 1 | Female | Male | .513 | 1.531 | .739 | -2.532 | 3.557 |
|  |  | Male | Female | -.513 | 1.531 | .739 | -3.557 | 2.532 |
|  | 2 | Female | Male | .513 | 1.531 | .739 | -2.532 | 3.557 |
|  |  | Male | Female | -.513 | 1.531 | .739 | -3.557 | 2.532 |
| 2 | 1 | Female | Male | 1.390 | 1.116 | .216 | -.830 | 3.610 |
|  |  | Male | Female | -1.390 | 1.116 | .216 | -3.610 | .830 |
|  | 2 | Female | Male | 1.390 | 1.116 | .216 | -.830 | 3.610 |
|  |  | Male | Female | -1.390 | 1.116 | .216 | -3.610 | .830 |
| Based on estimated marginal means | | | | | | | | |
| 1. Adjustment for multiple comparisons: Bonferroni.   Pain_Type1 = Social, Pain_Type2 = Physical | | | | | | | | |

| **Pairwise Comparisons** | | | | | | | |
| --- | --- | --- | --- | --- | --- | --- | --- |
| group | (I) Gender | (J) Gender | Mean Difference (I-J) | Std. Error | Sig.^a^ | 95% Confidence Interval for Difference^a^ | |
|  |  |  |  |  |  | Lower Bound | Upper Bound |
| 1 | Female | Male | .951 | 1.147 | .409 | -1.331 | 3.234 |
|  | Male | Female | -.951 | 1.147 | .409 | -3.234 | 1.331 |
| 2 | Female | Male | .951 | 1.147 | .409 | -1.331 | 3.234 |
|  | Male | Female | -.951 | 1.147 | .409 | -3.234 | 1.331 |
| Based on estimated marginal means | | | | | | | |
| a. Adjustment for multiple comparisons: Bonferroni. | | | | | | | |

DV 🡪 Pain Resonance

| **Tests of Within-Subjects Effects** | | | | | | | | | |
| --- | --- | --- | --- | --- | --- | --- | --- | --- | --- |
| Source | | Type III Sum of Squares | df | Mean Square | F | Sig. | Partial Eta Squared | Noncent. Parameter | Observed Power^a^ |
| Pain_Type | Sphericity Assumed | 20.612 | 1 | 20.612 | 11.619 | .001 | .122 | 11.619 | .921 |
|  | Greenhouse-Geisser | 20.612 | 1.000 | 20.612 | 11.619 | .001 | .122 | 11.619 | .921 |
|  | Huynh-Feldt | 20.612 | 1.000 | 20.612 | 11.619 | .001 | .122 | 11.619 | .921 |
|  | Lower-bound | 20.612 | 1.000 | 20.612 | 11.619 | .001 | .122 | 11.619 | .921 |
| Pain_Type * Group | Sphericity Assumed | 10.607 | 1 | 10.607 | 5.979 | .017 | .066 | 5.979 | .676 |
|  | Greenhouse-Geisser | 10.607 | 1.000 | 10.607 | 5.979 | .017 | .066 | 5.979 | .676 |
|  | Huynh-Feldt | 10.607 | 1.000 | 10.607 | 5.979 | .017 | .066 | 5.979 | .676 |
|  | Lower-bound | 10.607 | 1.000 | 10.607 | 5.979 | .017 | .066 | 5.979 | .676 |
| Pain_Type * Gender | Sphericity Assumed | 2.730 | 1 | 2.730 | 1.539 | .218 | .018 | 1.539 | .232 |
|  | Greenhouse-Geisser | 2.730 | 1.000 | 2.730 | 1.539 | .218 | .018 | 1.539 | .232 |
|  | Huynh-Feldt | 2.730 | 1.000 | 2.730 | 1.539 | .218 | .018 | 1.539 | .232 |
|  | Lower-bound | 2.730 | 1.000 | 2.730 | 1.539 | .218 | .018 | 1.539 | .232 |
| Error(Pain_Type) | Sphericity Assumed | 149.020 | 84 | 1.774 |  |  |  |  |  |
|  | Greenhouse-Geisser | 149.020 | 84.000 | 1.774 |  |  |  |  |  |
|  | Huynh-Feldt | 149.020 | 84.000 | 1.774 |  |  |  |  |  |
|  | Lower-bound | 149.020 | 84.000 | 1.774 |  |  |  |  |  |
| a. Computed using alpha = .05 | | | | | | | | | |

| **Tests of Between-Subjects Effects** | | | | | | | | |
| --- | --- | --- | --- | --- | --- | --- | --- | --- |
| Transformed Variable: Average | | | | | | | | |
| Source | Type III Sum of Squares | df | Mean Square | F | Sig. | Partial Eta Squared | Noncent. Parameter | Observed Power^a^ |
| Intercept | 150.568 | 1 | 150.568 | 37.644 | .000 | .309 | 37.644 | 1.000 |
| Group | 19.777 | 1 | 19.777 | 4.945 | .029 | .056 | 4.945 | .594 |
| Gender | 4.775 | 1 | 4.775 | 1.194 | .278 | .014 | 1.194 | .191 |
| Error | 335.983 | 84 | 4.000 |  |  |  |  |  |
| a. Computed using alpha = .05 | | | | | | | | |

| **Pairwise Comparisons** | | | | | | | | |
| --- | --- | --- | --- | --- | --- | --- | --- | --- |
| Pain_Type | Group1= LNG, GROUP2= HNG | (I) Gender | (J) Gender | Mean Difference (I-J) | Std. Error | Sig.^a^ | 95% Confidence Interval for Difference^a^ | |
|  |  |  |  |  |  |  | Lower Bound | Upper Bound |
| 1 | 1 | Female | Male | .087 | .401 | .830 | -.710 | .883 |
|  |  | Male | Female | -.087 | .401 | .830 | -.883 | .710 |
|  | 2 | Female | Male | .087 | .401 | .830 | -.710 | .883 |
|  |  | Male | Female | -.087 | .401 | .830 | -.883 | .710 |
| 2 | 1 | Female | Male | .623 | .380 | .104 | -.132 | 1.378 |
|  |  | Male | Female | -.623 | .380 | .104 | -1.378 | .132 |
|  | 2 | Female | Male | .623 | .380 | .104 | -.132 | 1.378 |
|  |  | Male | Female | -.623 | .380 | .104 | -1.378 | .132 |
| Based on estimated marginal means | | | | | | | | |
| 1. Adjustment for multiple comparisons: Bonferroni.   Pain_Type1 = Social, Pain_Type2 = Physical | | | | | | | | |

| **Pairwise Comparisons** | | | | | | | |
| --- | --- | --- | --- | --- | --- | --- | --- |
| Group1= LNG, GROUP2= HNG | (I) Gender | (J) Gender | Mean Difference (I-J) | Std. Error | Sig.^a^ | 95% Confidence Interval for Difference^a^ | |
|  |  |  |  |  |  | Lower Bound | Upper Bound |
| 1 | Female | Male | .355 | .325 | .278 | -.291 | 1.001 |
|  | Male | Female | -.355 | .325 | .278 | -1.001 | .291 |
| 2 | Female | Male | .355 | .325 | .278 | -.291 | 1.001 |
|  | Male | Female | -.355 | .325 | .278 | -1.001 | .291 |
| Based on estimated marginal means | | | | | | | |
| a. Adjustment for multiple comparisons: Bonferroni. | | | | | | | |

DV 🡪 Emotional Resonance

| **Tests of Within-Subjects Effects** | | | | | | | | | |
| --- | --- | --- | --- | --- | --- | --- | --- | --- | --- |
| Source | | Type III Sum of Squares | df | Mean Square | F | Sig. | Partial Eta Squared | Noncent. Parameter | Observed Power^a^ |
| Pain_Type | Sphericity Assumed | 22.085 | 1 | 22.085 | 1.964 | .165 | .023 | 1.964 | .283 |
|  | Greenhouse-Geisser | 22.085 | 1.000 | 22.085 | 1.964 | .165 | .023 | 1.964 | .283 |
|  | Huynh-Feldt | 22.085 | 1.000 | 22.085 | 1.964 | .165 | .023 | 1.964 | .283 |
|  | Lower-bound | 22.085 | 1.000 | 22.085 | 1.964 | .165 | .023 | 1.964 | .283 |
| Pain_Type * Group | Sphericity Assumed | 27.655 | 1 | 27.655 | 2.460 | .121 | .028 | 2.460 | .341 |
|  | Greenhouse-Geisser | 27.655 | 1.000 | 27.655 | 2.460 | .121 | .028 | 2.460 | .341 |
|  | Huynh-Feldt | 27.655 | 1.000 | 27.655 | 2.460 | .121 | .028 | 2.460 | .341 |
|  | Lower-bound | 27.655 | 1.000 | 27.655 | 2.460 | .121 | .028 | 2.460 | .341 |
| **Pain_Type * Gender** | Sphericity Assumed | 55.819 | 1 | 55.819 | 4.965 | .029 | .056 | 4.965 | .596 |
|  | Greenhouse-Geisser | 55.819 | 1.000 | 55.819 | 4.965 | .029 | .056 | 4.965 | .596 |
|  | Huynh-Feldt | 55.819 | 1.000 | 55.819 | 4.965 | .029 | .056 | 4.965 | .596 |
|  | Lower-bound | 55.819 | 1.000 | 55.819 | 4.965 | .029 | .056 | 4.965 | .596 |
| Error(Pain_Type) | Sphericity Assumed | 944.383 | 84 | 11.243 |  |  |  |  |  |
|  | Greenhouse-Geisser | 944.383 | 84.000 | 11.243 |  |  |  |  |  |
|  | Huynh-Feldt | 944.383 | 84.000 | 11.243 |  |  |  |  |  |
|  | Lower-bound | 944.383 | 84.000 | 11.243 |  |  |  |  |  |
| a. Computed using alpha = .05 | | | | | | | | | |

| **Tests of Between-Subjects Effects** | | | | | | | | |
| --- | --- | --- | --- | --- | --- | --- | --- | --- |
| Transformed Variable: Average | | | | | | | | |
| Source | Type III Sum of Squares | df | Mean Square | F | Sig. | Partial Eta Squared | Noncent. Parameter | Observed Power^a^ |
| Intercept | 2888.200 | 1 | 2888.200 | 53.900 | .000 | .391 | 53.900 | 1.000 |
| Group | 311.775 | 1 | 311.775 | 5.818 | .018 | .065 | 5.818 | .664 |
| Gender | .278 | 1 | .278 | .005 | .943 | .000 | .005 | .051 |
| Error | 4501.053 | 84 | 53.584 |  |  |  |  |  |
| a. Computed using alpha = .05 | | | | | | | | |

| **Pairwise Comparisons** | | | | | | | | |
| --- | --- | --- | --- | --- | --- | --- | --- | --- |
| Pain_Type | Group1= LNG, GROUP2= HNG | (I) Gender | (J) Gender | Mean Difference (I-J) | Std. Error | Sig.^a^ | 95% Confidence Interval for Difference^a^ | |
|  |  |  |  |  |  |  | Lower Bound | Upper Bound |
| 1 | 1 | Female | Male | -1.128 | 1.296 | .387 | -3.704 | 1.449 |
|  |  | Male | Female | 1.128 | 1.296 | .387 | -1.449 | 3.704 |
|  | 2 | Female | Male | -1.128 | 1.296 | .387 | -3.704 | 1.449 |
|  |  | Male | Female | 1.128 | 1.296 | .387 | -1.449 | 3.704 |
| 2 | 1 | Female | Male | 1.299 | 1.320 | .328 | -1.325 | 3.923 |
|  |  | Male | Female | -1.299 | 1.320 | .328 | -3.923 | 1.325 |
|  | 2 | Female | Male | 1.299 | 1.320 | .328 | -1.325 | 3.923 |
|  |  | Male | Female | -1.299 | 1.320 | .328 | -3.923 | 1.325 |
| Based on estimated marginal means | | | | | | | | |
| a. Adjustment for multiple comparisons: Bonferroni. | | | | | | | | |

| **Pairwise Comparisons** | | | | | | | |
| --- | --- | --- | --- | --- | --- | --- | --- |
| Group1= LNG, GROUP2= HNG | (I) Gender | (J) Gender | Mean Difference (I-J) | Std. Error | Sig.^a^ | 95% Confidence Interval for Difference^a^ | |
|  |  |  |  |  |  | Lower Bound | Upper Bound |
| 1 | Female | Male | .086 | 1.189 | .943 | -2.279 | 2.450 |
|  | Male | Female | -.086 | 1.189 | .943 | -2.450 | 2.279 |
| 2 | Female | Male | .086 | 1.189 | .943 | -2.279 | 2.450 |
|  | Male | Female | -.086 | 1.189 | .943 | -2.450 | 2.279 |
| Based on estimated marginal means | | | | | | | |
| a. Adjustment for multiple comparisons: Bonferroni. | | | | | | | |

DV 🡪 SOD Pain

| **Tests of Within-Subjects Effects** | | | | | | | | | |
| --- | --- | --- | --- | --- | --- | --- | --- | --- | --- |
| Source | | Type III Sum of Squares | df | Mean Square | F | Sig. | Partial Eta Squared | Noncent. Parameter | Observed Power^a^ |
| Pain_Type | Sphericity Assumed | 4.131 | 1 | 4.131 | 2.221 | .140 | .026 | 2.221 | .314 |
|  | Greenhouse-Geisser | 4.131 | 1.000 | 4.131 | 2.221 | .140 | .026 | 2.221 | .314 |
|  | Huynh-Feldt | 4.131 | 1.000 | 4.131 | 2.221 | .140 | .026 | 2.221 | .314 |
|  | Lower-bound | 4.131 | 1.000 | 4.131 | 2.221 | .140 | .026 | 2.221 | .314 |
| Pain_Type * Group | Sphericity Assumed | 14.180 | 1 | 14.180 | 7.624 | .007 | .083 | 7.624 | .779 |
|  | Greenhouse-Geisser | 14.180 | 1.000 | 14.180 | 7.624 | .007 | .083 | 7.624 | .779 |
|  | Huynh-Feldt | 14.180 | 1.000 | 14.180 | 7.624 | .007 | .083 | 7.624 | .779 |
|  | Lower-bound | 14.180 | 1.000 | 14.180 | 7.624 | .007 | .083 | 7.624 | .779 |
| Pain_Type * Gender | Sphericity Assumed | .055 | 1 | .055 | .030 | .864 | .000 | .030 | .053 |
|  | Greenhouse-Geisser | .055 | 1.000 | .055 | .030 | .864 | .000 | .030 | .053 |
|  | Huynh-Feldt | .055 | 1.000 | .055 | .030 | .864 | .000 | .030 | .053 |
|  | Lower-bound | .055 | 1.000 | .055 | .030 | .864 | .000 | .030 | .053 |
| Error(Pain_Type) | Sphericity Assumed | 156.227 | 84 | 1.860 |  |  |  |  |  |
|  | Greenhouse-Geisser | 156.227 | 84.000 | 1.860 |  |  |  |  |  |
|  | Huynh-Feldt | 156.227 | 84.000 | 1.860 |  |  |  |  |  |
|  | Lower-bound | 156.227 | 84.000 | 1.860 |  |  |  |  |  |
| a. Computed using alpha = .05 | | | | | | | | | |

| **Tests of Between-Subjects Effects** | | | | | | | | |
| --- | --- | --- | --- | --- | --- | --- | --- | --- |
| Transformed Variable: Average | | | | | | | | |
| Source | Type III Sum of Squares | df | Mean Square | F | Sig. | Partial Eta Squared | Noncent. Parameter | Observed Power^a^ |
| Intercept | 127.665 | 1 | 127.665 | 39.900 | .000 | .322 | 39.900 | 1.000 |
| Group | 1.764 | 1 | 1.764 | .551 | .460 | .007 | .551 | .114 |
| Gender | 2.236 | 1 | 2.236 | .699 | .406 | .008 | .699 | .131 |
| Error | 268.769 | 84 | 3.200 |  |  |  |  |  |
| a. Computed using alpha = .05 | | | | | | | | |

| **Pairwise Comparisons** | | | | | | | | |
| --- | --- | --- | --- | --- | --- | --- | --- | --- |
| Pain_Type | Group1= LNG, GROUP2= HNG | (I) Gender | (J) Gender | Mean Difference (I-J) | Std. Error | Sig.^a^ | 95% Confidence Interval for Difference^a^ | |
|  |  |  |  |  |  |  | Lower Bound | Upper Bound |
| 1 | 1 | Female | Male | .205 | .421 | .628 | -.632 | 1.041 |
|  |  | Male | Female | -.205 | .421 | .628 | -1.041 | .632 |
|  | 2 | Female | Male | .205 | .421 | .628 | -.632 | 1.041 |
|  |  | Male | Female | -.205 | .421 | .628 | -1.041 | .632 |
| 2 | 1 | Female | Male | .281 | .300 | .352 | -.316 | .877 |
|  |  | Male | Female | -.281 | .300 | .352 | -.877 | .316 |
|  | 2 | Female | Male | .281 | .300 | .352 | -.316 | .877 |
|  |  | Male | Female | -.281 | .300 | .352 | -.877 | .316 |
| Based on estimated marginal means | | | | | | | | |
| a. Adjustment for multiple comparisons: Bonferroni. | | | | | | | | |

| **Pairwise Comparisons** | | | | | | | |
| --- | --- | --- | --- | --- | --- | --- | --- |
| Group1= LNG, GROUP2= HNG | (I) Gender | (J) Gender | Mean Difference (I-J) | Std. Error | Sig.^a^ | 95% Confidence Interval for Difference^a^ | |
|  |  |  |  |  |  | Lower Bound | Upper Bound |
| 1 | Female | Male | .243 | .291 | .406 | -.335 | .821 |
|  | Male | Female | -.243 | .291 | .406 | -.821 | .335 |
| 2 | Female | Male | .243 | .291 | .406 | -.335 | .821 |
|  | Male | Female | -.243 | .291 | .406 | -.821 | .335 |
| Based on estimated marginal means | | | | | | | |
| a. Adjustment for multiple comparisons: Bonferroni. | | | | | | | |

DV 🡪 SOD Emotions

| **Tests of Within-Subjects Effects** | | | | | | | | | |
| --- | --- | --- | --- | --- | --- | --- | --- | --- | --- |
| Source | | Type III Sum of Squares | df | Mean Square | F | Sig. | Partial Eta Squared | Noncent. Parameter | Observed Power^a^ |
| Pain_Type | Sphericity Assumed | 193.234 | 1 | 193.234 | 19.143 | .000 | .186 | 19.143 | .991 |
|  | Greenhouse-Geisser | 193.234 | 1.000 | 193.234 | 19.143 | .000 | .186 | 19.143 | .991 |
|  | Huynh-Feldt | 193.234 | 1.000 | 193.234 | 19.143 | .000 | .186 | 19.143 | .991 |
|  | Lower-bound | 193.234 | 1.000 | 193.234 | 19.143 | .000 | .186 | 19.143 | .991 |
| Pain_Type * Group | Sphericity Assumed | 49.495 | 1 | 49.495 | 4.903 | .030 | .055 | 4.903 | .591 |
|  | Greenhouse-Geisser | 49.495 | 1.000 | 49.495 | 4.903 | .030 | .055 | 4.903 | .591 |
|  | Huynh-Feldt | 49.495 | 1.000 | 49.495 | 4.903 | .030 | .055 | 4.903 | .591 |
|  | Lower-bound | 49.495 | 1.000 | 49.495 | 4.903 | .030 | .055 | 4.903 | .591 |
| Pain_Type * Gender | Sphericity Assumed | .494 | 1 | .494 | .049 | .826 | .001 | .049 | .055 |
|  | Greenhouse-Geisser | .494 | 1.000 | .494 | .049 | .826 | .001 | .049 | .055 |
|  | Huynh-Feldt | .494 | 1.000 | .494 | .049 | .826 | .001 | .049 | .055 |
|  | Lower-bound | .494 | 1.000 | .494 | .049 | .826 | .001 | .049 | .055 |
| Error(Pain_Type) | Sphericity Assumed | 847.915 | 84 | 10.094 |  |  |  |  |  |
|  | Greenhouse-Geisser | 847.915 | 84.000 | 10.094 |  |  |  |  |  |
|  | Huynh-Feldt | 847.915 | 84.000 | 10.094 |  |  |  |  |  |
|  | Lower-bound | 847.915 | 84.000 | 10.094 |  |  |  |  |  |
| a. Computed using alpha = .05 | | | | | | | | | |

| **Tests of Between-Subjects Effects** | | | | | | | | |
| --- | --- | --- | --- | --- | --- | --- | --- | --- |
| Transformed Variable: Average | | | | | | | | |
| Source | Type III Sum of Squares | df | Mean Square | F | Sig. | Partial Eta Squared | Noncent. Parameter | Observed Power^a^ |
| Intercept | 4041.992 | 1 | 4041.992 | 137.014 | .000 | .620 | 137.014 | 1.000 |
| Group | 195.560 | 1 | 195.560 | 6.629 | .012 | .073 | 6.629 | .721 |
| Gender | 7.583 | 1 | 7.583 | .257 | .613 | .003 | .257 | .079 |
| Error | 2478.046 | 84 | 29.501 |  |  |  |  |  |
| a. Computed using alpha = .05 | | | | | | | | |

| **Pairwise Comparisons** | | | | | | | | |
| --- | --- | --- | --- | --- | --- | --- | --- | --- |
| Pain_Type | Group1= LNG, GROUP2= HNG | (I) Gender | (J) Gender | Mean Difference (I-J) | Std. Error | Sig.^a^ | 95% Confidence Interval for Difference^a^ | |
|  |  |  |  |  |  |  | Lower Bound | Upper Bound |
| 1 | 1 | Female | Male | .333 | 1.058 | .754 | -1.771 | 2.437 |
|  |  | Male | Female | -.333 | 1.058 | .754 | -2.437 | 1.771 |
|  | 2 | Female | Male | .333 | 1.058 | .754 | -1.771 | 2.437 |
|  |  | Male | Female | -.333 | 1.058 | .754 | -2.437 | 1.771 |
| 2 | 1 | Female | Male | .561 | .985 | .570 | -1.397 | 2.520 |
|  |  | Male | Female | -.561 | .985 | .570 | -2.520 | 1.397 |
|  | 2 | Female | Male | .561 | .985 | .570 | -1.397 | 2.520 |
|  |  | Male | Female | -.561 | .985 | .570 | -2.520 | 1.397 |
| Based on estimated marginal means | | | | | | | | |
| a. Adjustment for multiple comparisons: Bonferroni. | | | | | | | | |

| **Pairwise Comparisons** | | | | | | | |
| --- | --- | --- | --- | --- | --- | --- | --- |
| Group1= LNG, GROUP2= HNG | (I) Gender | (J) Gender | Mean Difference (I-J) | Std. Error | Sig.^a^ | 95% Confidence Interval for Difference^a^ | |
|  |  |  |  |  |  | Lower Bound | Upper Bound |
| 1 | Female | Male | .447 | .882 | .613 | -1.307 | 2.201 |
|  | Male | Female | -.447 | .882 | .613 | -2.201 | 1.307 |
| 2 | Female | Male | .447 | .882 | .613 | -1.307 | 2.201 |
|  | Male | Female | -.447 | .882 | .613 | -2.201 | 1.307 |
| Based on estimated marginal means | | | | | | | |
| a. Adjustment for multiple comparisons: Bonferroni. | | | | | | | |
