# Supplementary material for: I understand your pain but I do not feel it: lower affective empathy in response to others’ social pain in narcissism
Source: Front Psychol. 2024 Mar 15;15:1350133. doi: 10.3389/fpsyg.2024.1350133 (PMC10994002; doi:10.3389/fpsyg.2024.1350133)
Supplement: Supplementary Material 2: — Gender-Related Analyses. [file Data_Sheet_2.docx]

**Supplementary data file 2. Correlations between self-other distinction (within participants measure) and resonance measures**

Correlations were conducted using z-scores, combining emotion and pain into a single measure where relevant, i.e. for the within-participant and resonance measures. This process resulted in three variables for each group: within-participant ratings (primary self-other-distinction measure), resonance (emotion and pain for participants and targets), and Intensity (which compared participant arousal and intensity for target). Correlations are presented separately for the social and physical pain videos for the high and low narcissism groups.

**HNG**

**Social Pain**

| **Correlations** | | | | |
| --- | --- | --- | --- | --- |
|  | | SOD within Social | SOD resonance social | SOD intensity social |
| SOD within social | Pearson Correlation | 1 | .450^**^ | .358^*^ |
|  | Sig. (2-tailed) |  | .002 | .017 |
|  | N | 44 | 44 | 44 |
| SOD resonance social | Pearson Correlation | .450^**^ | 1 | .942^**^ |
|  | Sig. (2-tailed) | .002 |  | .000 |
|  | N | 44 | 44 | 44 |
| SOD intensity Social | Pearson Correlation | .358^*^ | .942^**^ | 1 |
|  | Sig. (2-tailed) | .017 | .000 |  |
|  | N | 44 | 44 | 44 |
| **. Correlation is significant at the 0.01 level (2-tailed). | | | | |
| *. Correlation is significant at the 0.05 level (2-tailed). | | | | |

**Physical Pain**

| **Correlations** | | | | |
| --- | --- | --- | --- | --- |
|  | | SOD within physical | SOD resonance physical | SOD intensity physical |
| SOD within physical | Pearson Correlation | 1 | .406^**^ | .354^*^ |
|  | Sig. (2-tailed) |  | .006 | .018 |
|  | N | 44 | 44 | 44 |
| SOD resonance physical | Pearson Correlation | .406^**^ | 1 | .906^**^ |
|  | Sig. (2-tailed) | .006 |  | .000 |
|  | N | 44 | 44 | 44 |
| SOD intensity physical | Pearson Correlation | .354^*^ | .906^**^ | 1 |
|  | Sig. (2-tailed) | .018 | .000 |  |
|  | N | 44 | 44 | 44 |
| **. Correlation is significant at the 0.01 level (2-tailed). | | | | |
| *. Correlation is significant at the 0.05 level (2-tailed). | | | | |

**LNG**

**Social Pain**

| **Correlations** | | | | |
| --- | --- | --- | --- | --- |
|  | | SOD within social | SOD resonance social | SOD intensity Social |
| SOD within social | Pearson Correlation | 1 | .580^**^ | .853^**^ |
|  | Sig. (2-tailed) |  | .000 | .000 |
|  | N | 43 | 43 | 43 |
| SOD resonance social | Pearson Correlation | .580^**^ | 1 | .479^**^ |
|  | Sig. (2-tailed) | .000 |  | .001 |
|  | N | 43 | 43 | 43 |
| SOD intensity Social | Pearson Correlation | .853^**^ | .479^**^ | 1 |
|  | Sig. (2-tailed) | .000 | .001 |  |
|  | N | 43 | 43 | 43 |
| **. Correlation is significant at the 0.01 level (2-tailed). | | | | |

**Physical Pain**

| **Correlations** | | | | |
| --- | --- | --- | --- | --- |
|  | | SOD within physical | SOD resonance physical | SOD intensity physical |
| SOD within physical | Pearson Correlation | 1 | .377^*^ | .820^**^ |
|  | Sig. (2-tailed) |  | .013 | .000 |
|  | N | 43 | 43 | 43 |
| SOD resonance physical | Pearson Correlation | .377^*^ | 1 | .456^**^ |
|  | Sig. (2-tailed) | .013 |  | .002 |
|  | N | 43 | 43 | 43 |
| SOD intensity physical | Pearson Correlation | .820^**^ | .456^**^ | 1 |
|  | Sig. (2-tailed) | .000 | .002 |  |
|  | N | 43 | 43 | 43 |
| *. Correlation is significant at the 0.05 level (2-tailed). | | | | |
| **. Correlation is significant at the 0.01 level (2-tailed). | | | | |
